# Marine Natural Products from the Russian Pacific as Sources of Drugs for Neurodegenerative Diseases

**DOI:** 10.3390/md20110708

**Published:** 2022-11-11

**Authors:** Yuri S. Khotimchenko, Denis N. Silachev, Vladimir L. Katanaev

**Affiliations:** 1 Institute of Life Sciences and Biomedicine, Far Eastern Federal University, 8 ul. Sukhanova, 690950 Vladivostok, Russia; 2 A.V. Zhirmunsky National Center of Marine Biology, Far Eastern Branch, Russian Academy of Sciences, 690950 Vladivostok, Russia; 3 Department of Functional Biochemistry of Biopolymers, A.N. Belozersky Research Institute of Physico-Chemical Biology, Moscow State University, 119992 Moscow, Russia; 4 Department of Cell Physiology and Metabolism, Translational Research Centre in Oncohaematology, Faculty of Medicine, University of Geneva, Rue Michel-Servet 1, 1211 Geneva, Switzerland

**Keywords:** marine natural products, neurodegenerative diseases, drug discovery, Russian Pacific

## Abstract

Neurodegenerative diseases are growing to become one of humanity’s biggest health problems, given the number of individuals affected by them. They cause enough mortalities and severe economic impact to rival cancers and infections. With the current diversity of pathophysiological mechanisms involved in neurodegenerative diseases, on the one hand, and scarcity of efficient prevention and treatment strategies, on the other, all possible sources for novel drug discovery must be employed. Marine pharmacology represents a relatively uncharted territory to seek promising compounds, despite the enormous chemodiversity it offers. The current work discusses one vast marine region—the Northwestern or Russian Pacific—as the treasure chest for marine-based drug discovery targeting neurodegenerative diseases. We overview the natural products of neurological properties already discovered from its waters and survey the existing molecular and cellular targets for pharmacological modulation of the disease. We further provide a general assessment of the drug discovery potential of the Russian Pacific in case of its systematic development to tackle neurodegenerative diseases.

## 1. Introduction: The Russian Pacific

**Figure 1:**
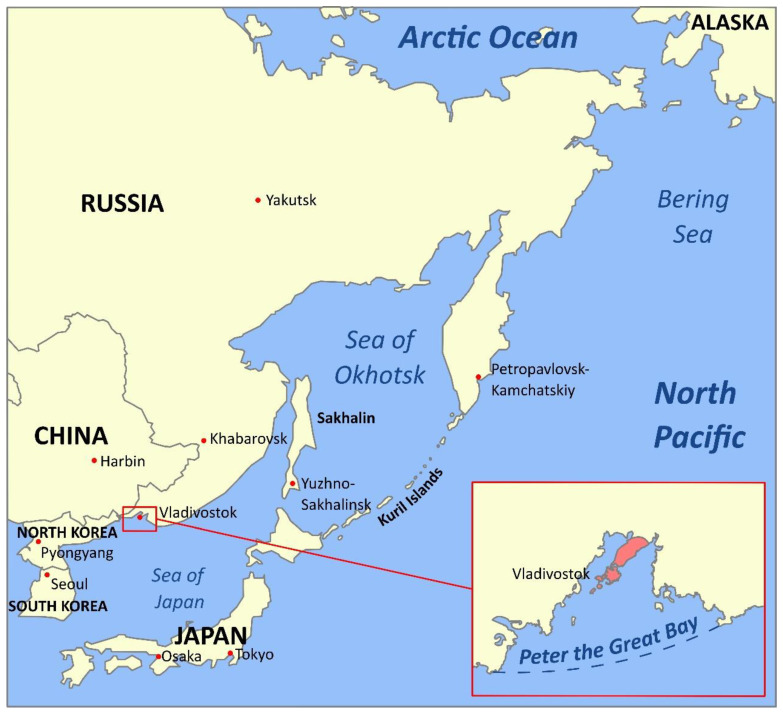
Map of the Russian Pacific and adjacent territories. The inset shows a higher magnification of the Peter the Great Gulf—a region of extreme biodiversity.

The Sea of Japan, the Sea of Okhotsk, the Bering Sea, the Pacific waters of the Kamchatka peninsula, and the Kuril Islands form a unique marine region—the Russian Pacific (Figure 1). It is adjacent to the Far Eastern borders of Russia from the shores of the Korean Peninsula to the Bering Strait, extending in the latitude-meridional direction for almost 5000 km from low arctic to subtropical climatic subzone [1]. Large hydrocarbon reserves on the shelf of the Far Eastern seas, gas and oil pipelines laid or projected along the sea coasts and depths, ports, and various coastal industries being (re)constructed—all gain this region a unique and increasing role in the Russian and Asian-Pacific economics [2]. The Far Eastern Seas and adjacent Pacific Ocean waters are Russia’s main fishing basins [3]. This region accounts for more than 2/3 of the total Russian catch of hydrobionts and concentrates nearly 90% of the entire resource base of the Russian fisheries [4]. According to the TINRO Center (Pacific branch of the Russian federal research institute of fisheries and oceanography), the total long-term biomass of the benthic macrofauna of the shelf and the continental slope (down to 2025 m) of the Russian Pacific is estimated at 38,640 thousand tons, of which 21,804 thousand tons are fish and cyclostomes and 16,896 thousand tons are invertebrates such as cephalopods, crabs, shrimps, gastropods and bivalves, sea urchins and sea cucumbers, jellyfish and ctenophores [5]. Of these reserves, the Sea of Okhotsk contains 22,543 thousand tons, the Bering Sea—8186, the Pacific waters of Kamchatka—3744, the oceanic waters of the Kuril Islands—2784, and the Sea of Japan—1383 thousand tons. By the biomass concentration, a different ranking emerges: the East Kamchatka region (64.5 tons/km^2^), the Kuril oceanic waters (27.1 tons/km^2^), the Bering Sea (24.5 tons/km^2^), the Sea of Okhotsk (16.5 tons/km^2^) and the Sea of Japan (11.7 tons/km^2^) [5]. The most populated areas in terms of biomass density are areas with narrow shelves, steep slopes, and difficult reliefs.

The diverse physical and geographical conditions of the Far Eastern seas, subtropical in the southern half of the Sea of Japan to the arctic in the northern parts of the Sea of Okhotsk and the Bering Sea, determine the exceptionally rich species composition in these marine areas. The Zoological institute of the Russian academy of sciences has counted 6900 species of invertebrates in the Russian Pacific, most of which are representatives of macrobenthos (crustaceans, mollusks, annelids) [6]. This figure exceeds the number of invertebrate species described in the coastal waters of all European countries (6500 species) and the Arctic sector of Russia (4800 species). The greatest biological diversity of invertebrates occurs in the Sea of Japan—2900 species or 42% of the total diversity of the region—followed by the Sea of Okhotsk (2700 species), the Bering Sea (2000 species), and the Chukchi Sea (950 species). It should be emphasized that coastal macrobenthos of the Russian Pacific is understudied, with some 40% more invertebrate species waiting to be discovered. The numbers presented do not cover deep-water macrobenthos and meiofauna [6].

The fauna of the Far Eastern seas presents high species diversity and high biomass density—features typical for seas where waters of various origins collide. The Sea of Japan is the most heterogeneous in terms of fauna, from pronounced cold-loving forms in the northwestern to clear subtropical fauna in the southeastern parts. Along the western coast of the Sea of Japan, subtropical species reach the Peter the Great Gulf (inset in Figure 1). In the eastern part of the sea, along the western coast of Japan, subtropical species become rapidly lost past the Noto Peninsula. Still, some penetrate much further north, up to Hokkaido. In the Sea of Okhotsk, warm-water forms are concentrated in the southwestern part, where they penetrate from the Sea of Japan with the warm Tsushima current, while some also reach the western coast of Kamchatka. The northern part of the Bering Sea is influenced by the fauna of the Arctic Ocean. The largest number of cold-loving Arctic-boreal forms in the Bering Sea is in the area of the Anadyr cold spot. At the same time, many warm-water Pacific species extend almost to the Bering Strait and partially penetrate the southern part of the Chukchi Sea [7].

The Peter the Great Gulf is the richest water area in terms of species and taxonomic diversity, not only in the Russian sector of the Sea of Japan but among all the seas of Russia. According to NSCMB—the A.V. Zhirmunsky National scientific centre of marine biology (Far Eastern branch of Russian academy of sciences)—to date, almost 4000 species (including 2600 invertebrates) representing 52 types and 105 classes of marine organisms have been described in the Peter the Great Gulf. The species of the Gulf are not only diverse but, in many ways, unique. Despite its small marine area size (about 9000 km^2^), boreal-arctic, boreal, low-boreal, subtropical, and even tropical species of invertebrates and fish coexist. About 640 species of marine unicellular and multicellular algae have been described here, including species used in the food and pharmaceutical industries (ahnfeltia, agarum, kelp); more than 70 species of marine fungi; about 100 species of coelenterates; 222 species of flatworms, 178 species of roundworms and 277 species annelids; more than 320 species of mollusks; 620 species of crustaceans; 74 species of echinoderms (including the famous Far Eastern trepang and the Japanese cucumaria); 8 species of sea urchins; more than 300 species of fish [8].

In addition to immigrant species, autochthonous speciation exists in all Far Eastern seas, represented in nearly all groups of invertebrates by entire ‘bushes’ of numerous, sometimes still insufficiently isolated, unique species, subspecies, and varieties. The centers of new speciation mostly reside in areas where waters of different origins meet. The most powerful centers of new speciation in the Sea of Okhotsk and the Bering Sea are the upper levels of the continental slope and in the Sea of Japan—the Peter the Great Gulf and the waters of South Sakhalin. Each sea has its own set of endemic forms producing three independent faunal provinces. However, a large number of forms are universal to the three seas given their common historical past, permitting the placement of the Far Eastern seas into the single Far Eastern subregion within the North Pacific Boreal Region [9].

A set of joint Russian–German expeditions dedicated to the deep-sea investigations of the Russian Pacific has brought a completely new understanding of the species diversity of the region. These expeditions were the combined effort of researchers from the NSCMB Center, V.I. Il’ichev Pacific oceanological institute, Far East geological institute (all three institutions belonging to the Far Eastern branch of Russian academy of sciences), P.P. Shirshov Institute of oceanology (Russian academy of sciences), Far Eastern federal university, St Petersburg university, German Centre for marine biodiversity research, Biozentrum Grindel and Zoological museum, Zoological state collection Munich, University of Geneva, University of Tokyo. The expeditions revealed an unexpectedly high species diversity of marine organisms at great depths, overturning the dogmatic views on deep-sea life and opening new perspectives in biological and bioorganic diversity studies. Indeed, with <100 species of macrofauna previously known from the deep waters of the Sea of Japan, the SoJaBio expedition (Sea of Japan Biodiversity Studies, 2010) identified 621 species at the depths of 500–3660 m, of which 203 species were completely new to science [10,11]. Similarly, about 300 species had been known to inhabit the open abyssal plain of the northwestern Pacific Ocean (depths of 5–6 km), while the KuramBio expedition (Kuril-Kamchatka Biodiversity study, 2012) collected >1780 species at depths of 4830–5830 m, of which ca. 60% completely new [12,13]. These and other deep-sea expeditions [14,15,16] to various regions of the Northwestern Pacific (the Sea of Japan, the basin of the northwestern Pacific Ocean, the Kuril-Kamchatka Trench, the Kuril Basin of the Sea of Okhotsk, etc.) showed that bathyal and abyssal regions of the Northwestern Pacific, as most likely other deep-water areas, is characterized by a very high diversity and abundance of benthic representatives of marine fauna [17,18,19]. Despite this remarkable progress in the studies of macrofauna, deep-sea microorganisms and representatives of the benthic meiofauna remain practically unexplored.

The largest academic institution in Russia—the A.V. Zhirmunsky National scientific center of marine biology of the Far Eastern branch of the Russian academy of sciences (NSCMB)—is the leader in the marine biology and biodiversity research of the Far Eastern waters. Its main research areas are fauna and flora, ecology and productivity of the biota of the Far Eastern seas and adjacent waters of the Pacific Ocean; deep-sea research of the World Ocean; protection, reproduction and rational use of marine biological resources; molecular genetics, biochemistry, and biotechnology of marine organisms; technologies for remote control and monitoring of biodiversity and marine biological resources in specially protected marine areas; medical and biological research including the pharmacology of bioactive compounds.

Another important academic player in the Russian Pacific is PIBOC—the Elyakov Pacific institute of bioorganic chemistry of the Far Eastern branch of the Russian academy of sciences. It is the leading Russian scientific institution in the fields of isolation and identification of marine natural compounds, bioorganic and organic chemistry, molecular immunology, microbiology, biochemistry, marine biology, and biotechnology. Thanks to the research projects of PIBOC, every year, the arsenal of marine natural compounds is replenished with 50–100 new representatives (alkaloids, polar steroids, glycosides, cerebrosides, terpenoids, quinonoid metabolites, and peptides), many of which exhibiting antitumor, immunomodulatory, antibacterial, antioxidant, anti-inflammatory and neuroprotective properties. Further, the PIBOC Collection of marine microorganisms (official acronym KMM) includes over 4000 axenic strains of marine bacteria and fungi [20].

Research teams from leading Russian universities (Lomonosov Moscow state university, St Petersburg university, Goldberg Research institute of pharmacology and regenerative medicine (Tomsk), as well as scientists from Germany, Switzerland, South Korea, Japan, and the USA, participate in joint research projects on biomedical activities of marine natural compounds from the Russian Pacific.

Earlier, we published a review on the multidimensional assessment of the anticancer potential of marine invertebrates in the Russian Pacific [21]. In the present paper, we offer a multi-level analysis of the pharmacological promise this marine region offers for the prevention and treatment of neurodegenerative diseases.

## 2. Neurodegenerative Diseases: Problems and Pharmacotherapeutic Targets

Neurodegenerative diseases (NDD) are the most common forms of neurological pathologies and have become one of the most serious medical and social problems modern society faces [22]. There are several reasons for the rise of the medical and social importance of these diseases. First, the number of people suffering from them grows steadily in absolute and relative terms; among the elderly, NDD already outnumbers cancer as the second (after cardiovascular diseases) leading cause of death. Accordingly, the costs of treatment, rehabilitation, and care for this category of patients also steadily increase, and economic calculations predict that in the near future, these costs will become unbearable for many national economies. Second, while NDD have traditionally been thought to affect older people, recent data show that these geriatric diseases can also affect the population in their 40s and 50s, and it is not certain that the process of ‘rejuvenation’ will stop there. Third, we must face the fact that, as of today, there are practically no drugs for the effective treatment of these pathologies. Clinical symptoms at the end of NDD development result from neuronal death, which is pharmacologically irreversible. Thus, the main focus of translational research in this direction aims at preventing and/or slowing down the progression of neurodegenerative processes.

NDD can be grossly divided into two categories: demyelinating (such as multiple sclerosis and peroxisomal leukodystrophies) and nondemyelinating ones (such as Alzheimer’s disease, Parkinson’s disease, Huntington’s disease, Niemann-Pick disease, and amyotrophic lateral sclerosis) [22,23]. According to the WHO, brain disorders with a vascular and/or neurodegenerative component affect one billion people worldwide. In most cases, the disease begins with dementia. Disability due to dementia increases dramatically with age, affecting 9 out of 1000 people in the 65–74 age group and 83 per 1000 in the population over 85 years of age. Alzheimer’s disease (AD) is the main cause of dementia in Western countries and accounts for 45–60% [24] to 60–80% of all cases of dementia [25]. Parkinson’s disease (PD) is the second most common neurodegenerative disorder after AD, affecting 2% of the world’s population over 60 years of age [22,24].

NDD is characterized by progressive degradation of synapses and axons, leading to neuronal death and disruption of interneuronal connections, and is manifested by profound disorders of sensory, motor, and cognitive processes, including vision, hearing, movement, speech and language, memory, and more [22,26]. An important morphological feature of these diseases is the atrophy of the gray matter of the cerebral cortex—the key player in higher brain functions. While AD is characterized by extensive degeneration of cholinergic neurons located in the septum and basal forebrain [27], PD is characterized by progressive degeneration of nigrostriatal dopaminergic neurons [28]. In addition to such specific features, NDD share overlapping and common neurodegeneration mechanisms, including neuroinflammatory, metabolic, neurovascular, and genetic factors.

We will not dwell on the clinical, pathomorphological, and pathobiochemical characteristics of NDD in detail since these provisions have been extensively covered in the literature. Instead, we will review some pharmacological targets in NDD.

### 2.1. Pharmacotherapeutic Targets

#### 2.1.1. Amyloid-β and the Regulatory Enzymes

**Figure 2:**
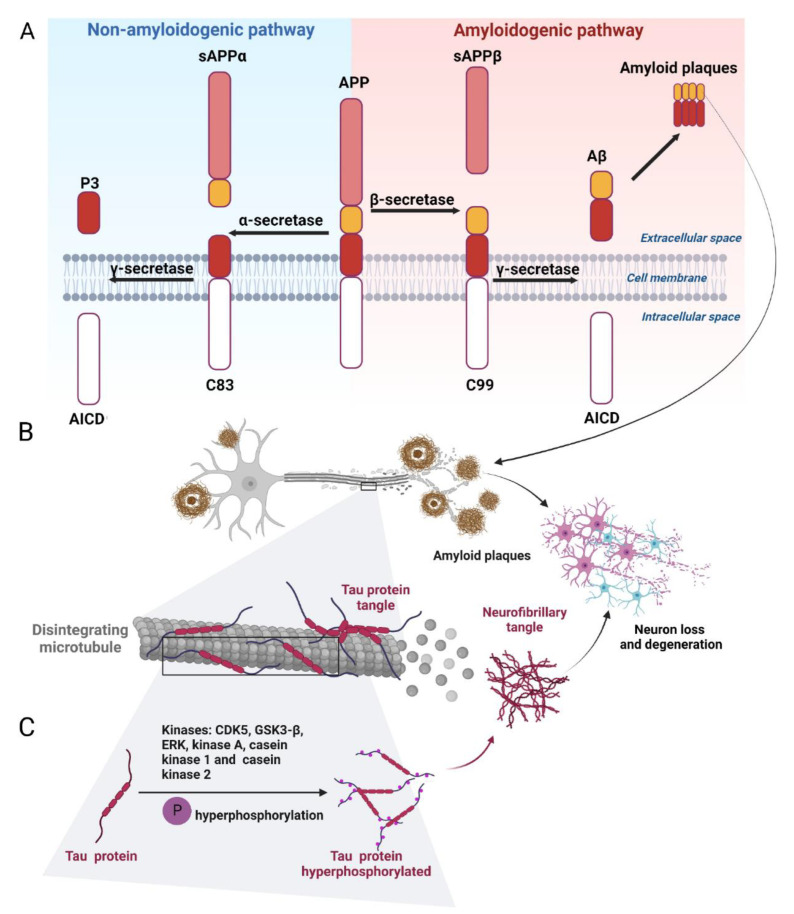
Beta-amyloid and tau protein in the pathogenesis of Alzheimer’s disease. (**А**) Diagram of the amyloid precursor protein (APP) processing pathway. AICD—APP intracellular fragment; P3—carboxyterminal fragment P3; sAPP-α and sAPPβ—soluble APP-fragments; C83—carboxyterminal fragment 83; C99—carboxyterminal fragment 99; Aβ—amyloid beta peptides (Aβ40/Aβ42). (**B**) Aβ fragments oligomerize and fibrillize to form amyloid plaques leading to AD pathology. (**C**) Hyperphosphorylation of tau protein causes microtubule depolarization; aggregates of tau oligomers assemble to form neurofibrillary tangles leading to loss of dendritic spines and deterioration of synaptic plasticity. Created with BioRender.com.

Amyloid plaques (neuritic or senile plaques) are globular deposits composed of extracellular agglomerates of amyloid-β protein (Aβ) derived from improper cleavage of the amyloid precursor protein (APP) [29,30]. APP is a type I transmembrane protein consisting of 695 to 770 amino acid residues, ubiquitously expressed, with particularly high levels of expression in the brain. It includes a 17-residues N-terminal signal peptide, a large ectodomain, a 23-amino acids hydrophobic transmembrane domain, and a 47-residues cytoplasmic domain. Three enzymes, α-, β-, and γ-secretases, are responsible for the proteolytic processing of APP. α-secretase cuts APP in the luminal region, producing a soluble fragment of the sAPPα ectodomain and an 83-residues C-terminal fragment (C83). Cleavage by α-secretase occurs between residues 16–17 of Aβ and interferes with Aβ formation, preventing its aggregation into plaques. Thus α-secretase is of a non-amyloidogenic activity [31]. The β-secretase (BACE1, β-site APP cleaving enzyme 1) cleaves the extracellular domain of APP at the N-terminal Aβ residue, 16 residues below the α-secretase cleavage site, generating a smaller soluble sAPPβ fragment and a larger membrane-bound APP C-terminal fragment consisting of 99 residues (C99) [32]. BACE1 overexpression increases Aβ production [33]. Notably, neuronal injury, inflammation, and oxidative stress all contribute to enhancing BACE1 expression and Aβ production [34]. The final cleavage of the C83 and C99 fragments is performed by γ-secretase, which chops away the C-terminus of both C83 and C99, releasing p3 and Aβ, respectively. This cleavage occurs in the transmembrane region of APP and produces two major Aβ variants: Aβ40 ending at residue 40 of APP and Aβ42 ending at the residue 42 (Figure 2A). Aβ42 is more aggregation-prone than Aβ40. The non-toxic monomers are then converted into toxic oligomers and deposited in the extracellular space as neuritic plaques and in the endoplasmic reticulum, endosomes, and trans-Golgi network of neurons in the form of Aβ fibrils [35,36]. These Aβ aggregates trigger several events, such as disruption of axonal transport, destabilization of microtubules, disruption of oxidative phosphorylation in mitochondria, and excessive production of reactive oxygen species (ROS) that lead to neuronal cell death (Figure 2B) [37,38].

Amyloidogenesis can be targeted by enhancing α-secretase [39,40] or by suppressing the β- and γ-secretases [31]. It should be noted that γ-secretase cleaves >50 substrates in addition to APP, mostly integral type I membrane proteins such as the Notch receptor. Likewise, BACE1 also processes substrates other than APP, including neuronal proteins important for synaptic plasticity [31]. Therefore, the search for molecules that inhibit or modulate specifically cleavages of APP by BACE1 and by γ-secretase remains topical [41,42]. Targeting of Aβ aggregation can also be envisioned, along with the acceleration of Aβ clearance. Thus, three potential strategies for anti-amyloid therapy emerge: inhibition of Aβ production, inhibition of Aβ aggregation, and promotion of Aβ removal.

#### 2.1.2. Tau Protein and Hyperphosphorylation of Tau

Tau is a microtubule-associated protein involved in the neuronal microtubule stability and regulation of synaptic functions [43]. Tau hyperphosphorylation promotes the formation of neurofibrillary tangles leading to the loss of dendritic spines and deterioration of synaptic plasticity [44,45]. Tau is phosphorylated by proline-directed protein kinases (PDPKs), such as cyclin-dependent kinase 5 (Cdk5), glycogen synthase kinase-3β (GSK3β), and extracellular signal-related protein kinase (ERK), or non-PDPKs such as protein kinase A (PKA), casein kinase 1 (CK1), and casein kinase 2 (CK2) (Figure 2C) [46,47]. Among these kinases, GSK3β plays a leading role in tau pathology [48] and has been a recognized target for AD treatment [49]. GSK3β is abundant in the brain and is associated with AD, PD, and Huntington’s disease (HD) [50,51,52], enlarging its pharmacological importance as an NDD target. Tau hyperphosphorylation is also regulated by phosphatases, mainly protein phosphatase 2A (PP2A). Memantine, an NMDA receptor antagonist, has been shown to increase PP2A activity and decrease tau phosphorylation both in vivo and in vitro [53].

Another target of relevance for tau phosphorylation is DYRK1A belonging to the family of dual-specificity tyrosine phosphorylation-regulated kinases. DYRK1A is expressed ubiquitously but is particularly high in the cerebellum, olfactory bulb, and hippocampus [54]. Increased DYRK1A activity also triggers Aβ peptide production through stimulation of the β/γ-secretase cleavage of APP [55,56]. In turn, Aβ further increases DYRK1A expression, thereby additionally stimulating the production of the neurotoxic Aβ [57]. Increased DYRK1A activity has been noted in patients with AD, PD, HD, Niemann-Pick disease, as well as Down’s syndrome [58].

#### 2.1.3. Glutamatergic System and Glutamatergic Neurotransmission

**Figure 3:**
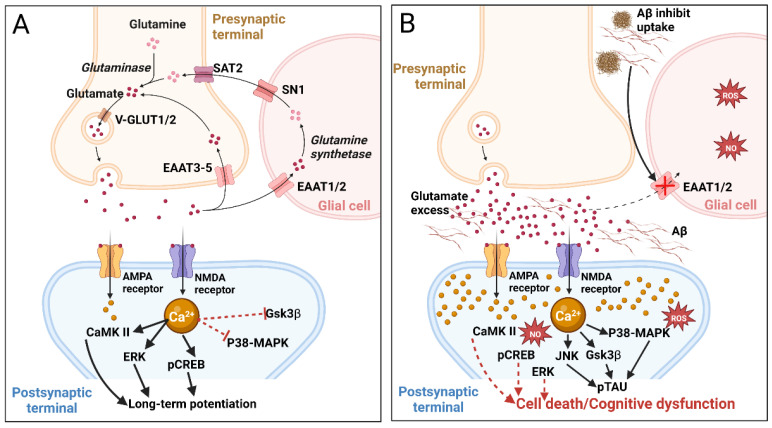
The role of the glutamatergic system in the development of neurodegenerative diseases. (**A**) Glutamate synthesis and cycling in normal brain. (**B**) Glutamate-mediated transmission in Alzheimer’s disease. VGlut1/2—vesicular glutamate transporter1/2; EAAT—excitatory amino acid transporter; SN1—glutamine transporter; SAT2—system A transporter; CaMKII—calcium calmodulin-dependent kinase II; ERK—extracellular signal-related kinase; pCREB—phosphorylated cyclic AMP response element binding protein; GSK3β—glycogen synthase kinase 3β; p38-MAPK—p38 mitogen-activated protein kinase; pTAU—hyperphosphorylated tau; NO—nitrogen oxide; ROS- reactive oxygen species. Created with BioRender.com.

More than 40% of neuronal synapses in the human brain are glutamatergic, using glutamate as the excitatory neurotransmitter and playing important roles in learning and memory. Neurons and glial cells produce glutamate from α-ketoglutarate. After release into the synaptic cleft and receptor activation, excess glutamate is taken up by excitatory amino acid transporters (EAAT) expressed by astrocytes (EAAT1/2) and neurons (EAAT3-5). The pharmacological relevance of these transporters is illustrated by the finding that LDN/OSU-0212320, a translational activator of EAAT2, reduced the number of Aβ plaques and improved cognitive functions in a mouse AD model [59]. In glial cells, glutamate is converted to inactive glutamine by glutamine synthetase; glutamine is released into the extracellular space, taken up by presynaptic neurons, and reduced back to glutamate by phosphate-activated glutaminase. Glutamate is transported to synaptic vesicles via the activity of vesicular glutamate transporters 1 and 2 (VGLUT1/2) (Figure 3A) [60,61].

Disruption of the glutamate/glutamine cycle can cause excitotoxicity by over-activating glutamate receptors, and the resultant excessive influx of Ca^2+^ can cause cell death (Figure 3B). Thus, the inactivation of EAAT1/2 or glutamine synthetase increases glutamate concentration in the synaptic cleft, causing overexcitation of the postsynaptic membrane and neurodegeneration. EAAT2 plays an important role in cognitive functions, and impairment/loss of its functions leads to cognitive disorders [59]. Similar perturbations of the glutamate/glutamine cycle may result from Aβ42 induction. Aβ deposition can activate lipid peroxidation in neuronal membranes, releasing 4-hydroxy-2-nonenal that promotes the generation of ROS [62]. Accumulation of Aβ plaques in the synapse and infiltration of the tau protein into dendritic spines also reduce glutamatergic synaptic transmission leading to cognitive impairments [63].

Post- and pre-synaptic ionotropic glutamate receptors include three species: N-methyl-D-aspartate (NMDA), α-amino-3-hydroxy-5-methyl-4isoxasolepropionic acid (AMPA), and kainate receptors, which provide rapid excitatory transmission. NMDA receptors are permeable to Na^+^, K^+^, and highly permeable to Ca^2+^, the latter acting as a second messenger stimulating intracellular signaling cascades involving Ca^2+^/calmodulin-dependent kinase II (CaMKII), ERK, and phosphorylation of cAMP response element-binding protein (CREB), which are involved in the induction of long-term potentiation (LTP) (Figure 3A) [64]. Elevated Ca^2+^ levels activate calpain I, phospholipases, and arachidonic acid metabolism, leading to the release of ROS and reactive nitrogen species that provoke the disintegration of the cytoskeleton and degeneration of neuronal membranes [65]. Ca^2+^-induced activation of protein kinases also leads to tau hyperphosphorylation [47]. NMDA receptors are heterotetramers composed of combinations of seven different subunits, localized both within the synapse and at extrasynaptic sites. Extrasynaptic NMDA receptors are involved in the regulation of Aβ production [66]. Thus, on the one hand, NMDA receptors support synaptic plasticity and survival of neurons, but on the other hand, their excessive activation leads to excitotoxicity, neurodegeneration, and cell death [67].

Accumulation of Aβ peptides and especially oligomeric Aβ in the hippocampus and cerebral cortex has been reported to suppress LTP, aberrating signaling cascades dependent on NMDA receptors and leading to long-term depression (LTD) [68]. Aβ peptides also enhance glutamate release by presynaptic neurons [69] and astrocytes [70]. Elevated glutamate activates extrasynaptic NMDA receptors leading to increased postsynaptic Ca^2+^ that triggers LTD and synaptic disruption. High [Ca^2+^] activates p38 mitogen-activated protein kinases (MAPKs), GSK3β, and c-Jun N-terminal kinase (JNK), all involved in cell death signaling and tau hyperphosphorylation (Figure 3B) [71]. Excess cytosolic Ca^2+^ is taken up by mitochondria triggering the formation of ROS and NO, inhibition of ATP synthesis, mitochondrial permeability, cytochrome c release, caspase activation, and ultimately apoptosis [72]. These findings highlight NMDA receptor antagonists as potential means to treat NDD. Indeed, memantine—a non-competitive NMDA receptor antagonist—reduces NMDA receptor-mediated excitotoxicity, improves cognition, suppresses tau phosphorylation, and slows disease progression [73,74]. Memantine (in 2003) and the combination of memantine with donepezil (in 2014) were approved by the Food and Drug Administration (FDA) to treat moderate to severe AD. To date, NMDA is considered one of the most popular pharmacological targets for the search for new anti-neurodegenerative drug molecules for AD correction [75].

AMPA receptors, consisting of GluA1, GluA2, GluA3, or GluA4 subunits, are expressed in both neuronal and glial cells. AMPA receptors maintain synaptic plasticity and are involved in learning and memory [76]. Aβ stimulates AMPA receptor endocytosis, resulting in synaptic depression [77].

Metabotropic glutamate receptors (mGluR1-mGluR8) are G-protein coupled receptors (GPCR) and provide fine-tuning in the synapse. mGluR5 is suspected to be involved in the pathogenesis of AD, PD, and HD; mGluR5 likely mediates the transmission of synaptotoxic signals induced by Aβ42 oligomers. The damaging effects of Aβ oligomers on mGluR5 include receptor overactivation, accumulation of intracellular Ca^2+^, receptor clustering, and synaptic disruption [78]. Genetic ablation of mGluR5 suppresses excitotoxic degeneration, thereby exerting a neuroprotective effect [79]. It has also been shown that activation of postsynaptic mGluR3 enhances neuroprotection by scavenging radicals [80]. Activation of mGluR2 increases neuronal vulnerability to Aβ. In contrast, dual activation of mGluR2 and mGluR3 protects against Aβ-induced neurotoxicity, which is associated with the release of TGF-β1 providing clearance of Aβ peptides and stimulating synaptic plasticity [81,82]. Activation of mGluR3 in astrocytes promotes non-amyloidogenic APP cleavage, with an increase in sAPPα and suppression of BACE1 expression [83]. Inhibition of mGluR2 has been shown to improve memory in rodent models of AD [84], and stimulation of mGluR2 causes TNF-α mediated activation of TNF receptor 1 and caspase-3, leading to microglial neurotoxicity [85]. Thus, mGluR2 and mGluR3 can be considered targets for searching for new anti-neurodegenerative compounds, and mGluR2 blockade and mGluR3 activation can be used as a strategy for the treatment of AD [86].

mGluR5 couples to the heterotrimeric G-protein Gαq/11; activation of the latter leads to an increase in intracellular [Ca^2+^], excess of which is a mechanistic input into many NDD. Genetic deletion of the mGluR5 abolishes cognitive decline in a mouse AD model [79]. Selective blockade of the mGluR5 with a negative allosteric modulator 2-chloro-4-[2-[2,5-dimethyl-1-[4-(trifluoromethoxy)phenyl]imidazol-4-yl]ethynyl]pyridine improved cognitive function in these mice [87], while another selective mGluR5 blocker 3-((2-methyl-1,3-thiazol-4-yl)ethynyl)pyridine restored learning and memory deficits in the AD mice by eliminating synaptic dysfunction [79,88]. Similarly, a silent allosteric mGluR5 modulator BMS-984923 restored memory deficits in this mouse model by prevention of the mGluR5-PrPc interaction and Aβ-induced pathological signaling [88]. mGluR5 also regulates the release of inflammatory factors and ATP in microglial cells and astrocytes [89]. LY341495, a nonselective group I/II mGluR antagonist, improved synaptic plasticity and abolished Aβ-induced LTD [90]. Finally, a non-competitive mGluR5 antagonist, SIB1757, prevented the Aβ-induced decrease in the number of NMDA receptors during neuronal pretreatment [78]. These data identify mGluR5 as the desired target in NDD. Interestingly, mGluR2/3 antagonists also displayed pro-cognitive effects in mice in the Morris water maze test [91], novel recognition test [92], and social recognition test [93].

#### 2.1.4. Cholinergic System and Dysfunction

Cholinergic neurons in the CNS regulate functions such as memory, attention, learning, and the sleep cycle [94]. Acetylcholine (ACh) is synthesized from choline and acetyl-CoA in the cytosol of presynaptic cholinergic neurons and stored in synaptic vesicles that undergo exocytosis upon neuronal depolarization. Upon release into the synaptic cleft, ACh binds to its receptors, including both ionotropic nicotinic (nAChR) receptors and metabotropic muscarinic (mAChR) GPCRs. nAChRs present in the CNS is mainly expressed presynaptically, where they regulate the release of other neurotransmitters. Stimulation of mAChRs usually results in excitation, especially in the cerebral cortex. Stimulation of ACh receptors is rapidly terminated by acetylcholinesterase (AChE), located in the synaptic cleft, and functionally associated with the postsynaptic membrane and hydrolyzing ACh to acetate and choline. The reuptake of choline by the presynaptic neuron is performed by choline carriers [95]. Dysfunction/dysregulation of nAChRs can be seen in various neurological and neuropsychiatric diseases and conditions, including chronic pain syndromes, schizophrenia, AD, PD, depression, etc. [94,96].

**Figure 4:**
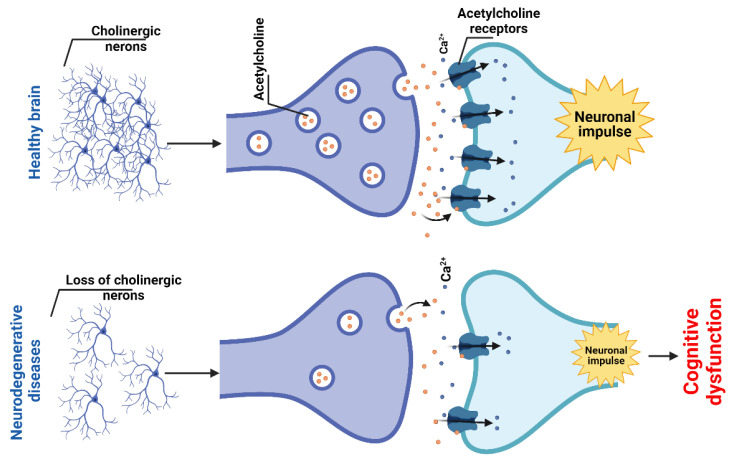
Schematic representation of the cholinergic hypothesis of neurodegenerative diseases. Cognitive dysfunction may be caused by a loss of cholinergic neurons in the septum and basal forebrain and a change in acetylcholine transmission. Created with BioRender.com.

According to the cholinergic hypothesis of dementia, the loss of cholinergic neurons in the septum and basal forebrain and the resulting loss of cortical cholinergic activity lead to cognitive decline in patients with AD [97] (Figure 4). However, these changes likely occur only in the advanced stages of the disease. Postmortem brains of dementia patients have revealed a decrease in choline acetyltransferase (ChAT) activity, which is likely to reflect a reduced synthesis of ACh [98]. It has been shown that the blockade of mAChRs leads to cognitive dysfunction resembling that in elderly patients. Conversely, cholinomimetics have been found to play a beneficial role in the treatment of cognitive dysfunction associated with AD [99]. Although cholinergic denervation is recognized as a pathological feature of AD, in vivo neuroimaging studies have also revealed a loss of cerebral cholinergic markers in parkinsonian dementia that is similar to or more severe than in prototypical AD [100].

AChE inhibitors are in use to treat mild and moderate forms of AD [101]. The FDA has approved four such drugs: tacrine (1993, no longer prescribed due to hepatotoxicity), donepezil (1996), rivastigmine (2000), and galantamine (2001). The first indication for AChE inhibitors was AD. Still, later other forms of dementia and CNS disorders were added, such as mild cognitive impairment, dementia with Lewy bodies, PD, Down’s syndrome, vascular dementia, and Korsakoff disease [102]. AChE rapidly hydrolyses Ach and is thus a key enzyme for the termination of neurotransmission; its activity is >100 times higher than that of butyrylcholinesterase (BChE) [103]. At advanced stages of AD, a decrease in brain AChE activity has been seen, accompanied by significantly increased levels and activity of BChE [104], which provokes further ACh deficiency and loss of cognitive functions [105]. There is a direct link between the cholinergic and amyloid pathways where Aβ deposition is accelerated by AChE. In AD, cholinergic neurons in the brain accumulate phosphorylated tau proteins and aggregated Aβ peptides and exhibit increased AChE activity early in the disease. These mechanisms lead to ACh deficiency and gradual loss of brain neurons. Against this background, AChE and BChE inhibitors prolong acetylcholine activity in brain synapses and may improve the cognitive and functional capabilities of patients with AD [106]. Thus, AChE and BChE, as well as nAChRs, are targets for pharmacotherapy in NDD, and marine compounds have been identified as agents acting at these targets [107,108,109].

#### 2.1.5. Neuroinflammation

**Figure 5:**
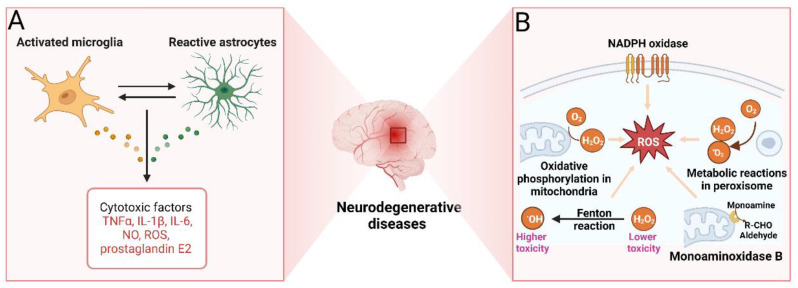
Neuroinflammation (**A**) and oxidative stress (**B**) are key pathogenic mechanisms involved in neurodegenerative diseases. (**A**) Reactive astrocytes and activated microglia produce cytotoxic factors that negatively affect neurodegenerative diseases. (**B**) Main sources of ROS production in cells. TNFα—tumor necrosis factor α; IL-1β—interleukin-1β; IL-6—interleukin-6; NO—nitrogen oxide; ROS—reactive oxygen species. Created with BioRender.com.

Neuroinflammation plays a key role in the onset and progression of NDD, but physiologically it is a protective reaction of the brain to damaging factors. However, an excessive inflammatory response becomes damaging and eventually leads to neuronal cell death [110]. Resident immune cells are represented in the CNS by microglia that play a major role in the neuroinflammatory response. Upon brain injury or exposure to pathogens, microglia are activated to initiate their phagocytic functions and release pro-inflammatory and cytotoxic factors such as IL-1β, IL-6, NO, ROS, TNF-α, and prostaglandin E2 (PGE2) (Figure 5A) [111]. The burst of pro-inflammatory cytokines promotes Aβ and tau accumulation in nearby neurons [112]. Neurons damaged by Aβ enter a vicious cycle in which the production of pro-inflammatory cytokines triggers the release of neurotoxic Aβ, which in turn initiates reactive microglia to release additional cytokines [113]. This mechanism highlights chronic irreversible neuroinflammation as a leading factor in the neurodegenerative process [114]. Currently, a number of kinases involved in neuroinflammation are considered potential pharmacotherapeutic targets, in particular, protein kinase C (PKC), CaMKII, MAPKs, JNK, GSK3β, or Janus kinase (JAK). Inhibitors of these kinases may be useful in a number of NDD, chronic pain, and epilepsy, as well as in ischemia [115,116,117,118,119].

In AD, microglia uptake and proteolyze neurotoxic Aβ [120]. However, over time, the efficiency of microglial clearance decreases, leading to increased amyloid deposition and release of pro-inflammatory cytokines, ultimately culminating in neuronal death. Thus, the microglial response is neuroprotective in the early stage but neurotoxic in the late stage of the disease. Accordingly, inhibition of the inflammatory response of microglial cells and protection of neuronal cells could potentially prevent the development of AD. A potential therapeutic strategy to treat the disease is thus the search for agents inhibiting microglial activation and controlling systemic inflammation [120].

Nuclear factor kappa B (NF-κB) plays an important role in neuroinflammation-mediated AD. As a major regulator of inflammatory gene transcription, NF-κB is elevated in the brains of Alzheimer’s patients [121]. Increased NF-κB signaling has been shown to upregulate BACE1 expression and thus contribute to disease development [122]. NF-κB is a negative regulator of Nrf2 [123]—a transcription factor orchestrating the antioxidant response mechanisms [124]. Given the reduced levels of Nrf2 in the hippocampus of AD patients, pharmacological Nrf2 activators might become desired agents to treat the disease [124]. NF-κB is also implicated in the pathophysiological mechanisms of PD: inflammation, mitochondrial dysfunction, synapse loss, and misfolded α-synuclein aggregation. Accumulation of α-synuclein induces NF-κB activation in neurons, promoting apoptosis and subsequent neuronal death. Misfolded α-synuclein released from degenerated neurons activates a number of signaling pathways in glial cells leading to NF-κB activation and production of pro-inflammatory cytokines, which exacerbate the neurodegenerative process. At the same time, NF-κB is necessary for the survival of neurons, in particular for the normal functioning of their mitochondria. Therefore, novel compounds capable of modifying NF-κB-related pathways are needed to prevent neuronal death [125].

#### 2.1.6. Oxidative Stress

Oxidative stress is defined as cell damage by ROS, and mitochondrial dysfunction is the main culprit in oxidative cell damage (Figure 5B) [126,127]. Aberrations in the glutathione metabolism represent another mechanism of oxidative damage in cells [128]. Finally, oxidative stress can be induced by the accumulation of iron that can react with hydrogen peroxide, producing more toxic hydroxyl and perhydroxyl radicals and leading to cell damage [129]. Brain cells consume large amounts of oxygen, inevitably exposing them to the risk of the formation of active forms of oxygen. In case of a shortage of agents normally converting reactive oxygen/nitrogen forms into safe inactive compounds, risks of oxidative stress become apparent. The situation is aggravated by the high brain content of polyunsaturated fatty acids easily oxidized to form toxic derivatives [130]. Further, the blood-brain barrier (BBB) limits the supply of natural antioxidants such as vitamin E to the brain, increasing the brain’s susceptibility to free radicals [131]. In AD, oxidative stress is one of the key drivers of pathogenesis. It leads to the disruption of normal APP processing and formation of Aβ42; accumulation of neurofibrillary tangles is also associated with oxidative stress [132]. Oxidative stress is also involved in the development of other NDD [133,134,135]. Therefore, compounds with antioxidant activities might be useful in preventing and treating NDD [136,137]. Antioxidant protection can be achieved by directly removing ROS, neutralizing active oxygen/nitrogen by interacting with their unpaired electrons, inhibiting ROS production, and binding metal ions that are necessary for ROS catalysis [138].

#### 2.1.7. α-Synuclein

Alpha-synuclein (α-syn) is a physiological regulator of neurotransmitter release. In the case of PD, intraneuronal inclusions called Lewy bodies are formed in the substantia nigra. Lewy bodies are composed of abnormal α-syn that is nitrated, phosphorylated, abnormally folded, and aggregated. In addition to Lewy bodies, abnormal neurites containing granular material and α-syn filaments (Lewy neurites) are also found in the substantia nigra of PD patients [139]. A pharmacological decrease in the amount of α-syn in the brain is attractive for disease treatment [140].

#### 2.1.8. Monoaminoxidase B

Monoamine oxidase (MAO) catalyzes the oxidative deamination of primary, secondary, and tertiary amines, including neurotransmitter monoamines, and plays an important role in the inactivation of catecholamines and 5-hydroxytryptamine [141]. The enzyme exists in two isoforms: MAO-A and MAO-B. While selective MAO-A inhibitors have a predominantly antidepressant effect, selective MAO-B inhibitors are intended for the treatment of PD and AD by replenishing dopamine deficiency and inhibiting the formation of Aβ, respectively [142,143]. Screening studies use MAO as a target in the search for new anti-NDD compounds, including those of marine origin [144,145].

#### 2.1.9. Adenosine Receptors

Adenosine is a neuromodulator that coordinates the functions of neurotransmitters responsible for motor functions and emotions. There are four types of adenosine receptors, A1, A2A, A2B, and A3, all GPCRs. The A1 and A3 receptors are G_i/o_-, and the A2A/B–G_s_-coupled. A2A receptors are also involved in inflammation processes through induction of TNF-α, as well as macrophage inflammatory proteins (MIP)-1α, MIP-1β, MIP-2α and MIP-3α [146]. Adenosine and dopamine D2 receptors have opposite effects on cAMP production, so inhibition of A2A receptors may enhance dopamine D2 receptor signaling [147]. As a result, inhibition of the A2A adenosine receptor has become an attractive strategy for the development of non-dopamine therapies for PD; marine compounds are interesting in this regard [148].

#### 2.1.10. The JNK Pathway

The JNK pathway has already been introduced above as one of the cellular mechanisms implicated in neuroinflammation [71]. Excessive activation of this pathway leads to neuronal cell death [149]. JNK includes several isoforms encoded by three genes: JNK1 (4 isoforms), JNK2 (4 isoforms), and JNK3 (2 isoforms) [150]. Of the resulting ten isoforms, JNK3α1 and JNK1α1 are the major JNK isoforms expressed in the brain [151]. Downstream of JNK, the transcription factors c-Jun, ATF2, SP1, NFATc2, and NFATc3 are substrates for phosphorylation by activated JNK. Dephosphorylation of JNK by a dual specificity phosphatase (DUSP1/MKP1) results in JNK deactivation. It has been shown that various biological markers of neurodegeneration, such as cytokines, ROS, or Aβ peptide, activate the JNK-c-Jun cascade and are involved in the maturation of AD neurofibrillary tangles [152]. JNK activation has also been observed in cultures of primary cortical neurons incubated with Aβ peptides and in transgenic mice with mutant presenilin, and finally, in AD brains [153]. Activation of the JNK signaling pathway has also been observed in the AD model in rats induced by Aβ25-35 [154]. Reciprocally, inhibition of JNK signaling reduces Aβ25-35-induced toxicity in primary neurons [155]. The accelerated aging SAMP-8 mice represent a spontaneous animal model carrying behavioral and histopathological features of AD, namely cognitive impairment, loss of neurons and dendrites, gliosis, cholinergic deficit in the forebrain, β-amyloid deposits resembling senile plaques, and hyperphosphorylation of tau [156]. In this model, cognitive deficits have been found to be associated with activation of the JNK pathway [157]; restoration of the cognitive capacities and reduced tau phosphorylation have been seen in these mice through inhibition of JNK by docosahexaenoic acid [158]. Cumulatively, these data provide ground for including JNK in the list of therapeutic targets for NDD [159].

#### 2.1.11. Autophagy

Autophagy is one of the processes by which cells break down unwanted molecules. In NDD, the autophagy-lysosome pathway is disrupted due to the sheer quantity of misfolded, mutated, and/or aggregated proteins that neurons must cope with. With an increased load of misfolded proteins, this pathway fails its function and becomes defective, aggravating protein aggregation [160]. Autophagy defects have been seen in many NDD, associated with senile plaques in AD, Lewy bodies in PD, nuclear and cytoplasmic inclusions containing aggregates of polyQ-expanded huntingtin in HD, aggregation of superoxide dismutase (SOD) in bunina bodies in ALS [161,162]. Marine compounds such as coibamide A isolated from marine cyanobacteria and papuamine isolated from the sea sponge *Haliclona* have been shown to induce autophagy and reduce levels of misfolded and aggregated proteins [163,164]. These findings identify neuronal autophagy as another relevant target for the development of therapeutic agents for neurodegeneration [165,166].

#### 2.1.12. Matrix Metalloproteinases

Active participants in neuroinflammation and NDD are matrix metalloproteinases (MMPs), enzymes that can degrade most components of the extracellular matrix, participating in physiological tissue remodeling, wound healing, and inflammatory processes [167]. In the CNS, MMPs regulate such physiological events as neurogenesis, angiogenesis, myelinogenesis, myelin turnover, axonal growth and guidance, synaptic plasticity, learning and memory, cell-fate specification, and signaling, and in neurodevelopmental processes involving ECM remodeling such as neuronal progenitor migration, axonal growth, myelinogenesis, and angiogenesis [168]. MMPs are also involved in pathological events in the CNS, such as disruption of BBB, neuronal death, axonal death, cytotoxicity, oxidative stress, demyelination, and inflammation [169]. Brain injury, hypoxia, ischemia, infections, and neurodegeneration trigger neuroinflammation, which is associated with increased production of MMPs [170]. In the initial phases of the acute inflammatory process of hypoxia-ischemia, free radicals and MMPs attack the proteins of the tight junctions and components of the basal lamina surrounding brain blood vessels, causing edema, hemorrhage, and cell death [171]. MMP-2, -3, and -9 are able to increase BBB permeability, and MMPs inhibitors can reduce BBB damage [172]. In rats subjected to ischemia, MMP-12 is activated, and its suppression protects BBB integrity [173].

In AD, deposition of Aβ activates microglia and astrocytes that start producing MMPs. In vitro, Aβ-exposed astrocytes secrete MMP-2, MMP-3, and MMP-9 [174]. MMP-9 is also expressed in neurons and is found in the plasma of AD patients [175,176]. In addition, MMP-3 expression is found in hippocampal neurons around amyloid plaques [177]. Increased MMP levels in AD patients exacerbate the inflammatory response and further promote neuronal death [178]. Interestingly, MMP-9 inhibition has been shown to promote Aβ elimination via BBB [179]. The contribution of MMPs to the pathophysiology of PD includes microglial activation, inflammation, dopaminergic apoptosis, BBB disruption, and α-syn cleavage [180,181,182].

Since excessive production of MMPs is implicated in the pathology of many inflammatory and malignant diseases, MMPs have become targets for drug discovery. While the first MMP inhibitors were synthetic, in recent years, increasing attention has been paid to the search for natural inhibitors, including those of marine origin, as MMP inhibitors in NDD [169].

#### 2.1.13. Neurotrophic Factors

Neurotrophic factors (NTFs) are small proteins that play important roles in the development and maintenance of structures of both the central and peripheral nervous systems, regulating growth, differentiation, synaptogenesis, and survival of neurons, ultimately controlling memory and cognition. NTFs can be divided into four families: neurotrophins, neurokines, the glial cell line-derived NTF family, and the cerebral dopamine NTF/mesencephalic astrocyte-derived NTF family. The most studied NTFs, such as brain-derived neurotrophic factor (BDNF), nerve growth factor (NGF), glial cell line-derived neurotrophic factor (GDNF), neurotrophin 3 and neurotrophin 4/5, and others, are considered pharmacological targets in NDD [183,184]. It has been shown that many NTFs are synthesized in precisely those areas of the brain that are affected by NDD, especially at an early stage of the disease [185].

According to the neurotrophic hypothesis, AD may be caused by a deficiency of expression of NTFs or their receptors [184]. In AD patients, increased NGF and decreased BDNF in the hippocampus and in the neocortex have been reported [186]. NTF signaling is seriously impaired in AD, which may be associated with cognitive decline [184]. Reduced levels of BDNF, NGF, and GDNF have been reported in patients with moderate AD and mild cognitive impairment [187]. GDNF is critical for the survival and maintenance of midbrain dopaminergic neurons [188] and it has been hypothesized that reduced GDNF levels cause excessive release of glutamate, leading to CNS excitotoxicity that precedes dopaminergic degeneration [189]. Moreover, GDNF has been found to prevent oxidative stress in both neurons and glial cells [190] and is able to protect substantia nigra dopamine neurons from the toxic effects of 6-hydroxydopamine [189].

Noteworthy, dopaminergic degeneration is significantly associated with PD and AD, and pharmacological interventions aimed at increasing dopaminergic transmission in the hippocampus and cerebral cortex have been shown to improve synaptic functions and memory deficits in AD patients [191]. The use of neurotrophic factors themselves as therapeutic agents is complicated by their poor ability to penetrate through BBB. This problem should be solved by searching for pharmacological agents that can induce the synthesis and release of endogenous NFTs in the corresponding areas of the brain or activate certain NTF receptors.

#### 2.1.14. Peroxisome Proliferator-Activated Receptor Gamma Coactivator-1 Alpha (PGC-1α)

PGC-1α is a transcriptional coactivator and metabolic regulator initially identified in brown adipose tissue as a peroxisome proliferator-activated receptor γ (PPARγ) coactivator in the thermogenic response to cold. Subsequent work has shown that PGC-1α is involved in NDD by activating mitochondrial biogenesis and respiration [192]. PGC-1α induces expression of ROS scavenging enzymes (glutathione peroxidase-1 (GPX1), catalase (CAT), and SOD) and thus reduces oxidative stress [193]. In PGC-1α knockout mice, exposure to 1-methyl-4-phenyl-1,2,3,6-tetrahydropyridine resulted in degeneration of nigral dopaminergic neurons, suggesting that PGC-1α has significant neuroprotective activity. In addition, an increase in PGC-1α levels significantly protected nerve cells from oxidative stress and cell death, and PGC-1α activation counteracted mutant α-syn or the pesticide rotenone in the loss of dopaminergic neurons [194,195]. Upregulation of neuronal PGC-1α may prevent mitochondrial dysfunction in neurodegenerative encephalopathy models in vitro and in vivo [196]. Since impaired mitochondrial function is at the basis of the pathogenesis of almost all NDD, and given the strong evidence for the involvement of PGC-1α in major NDD including AD, PD, HD, and ALS [197], PGC-1α can be considered as a pharmacotherapeutic target in NDD and screening for new anti-neurodegenerative agents [198].

#### 2.1.15. The Microbiota-Gut-Brain Axis

**Figure 6:**
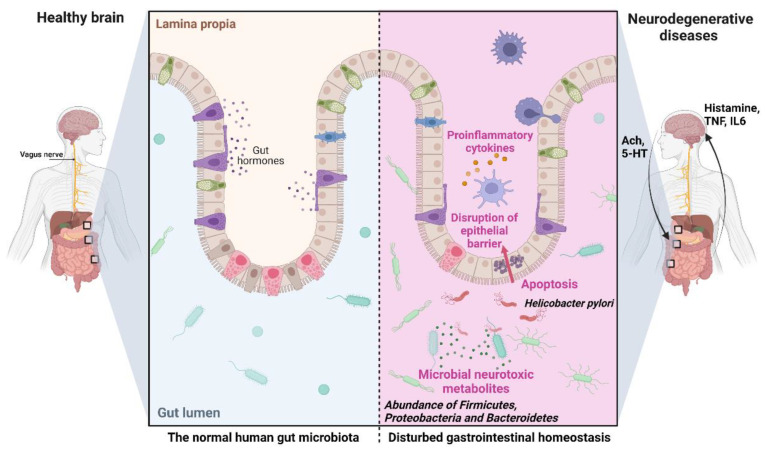
Schematic diagram shows the role of the microbiota-gut-brain axis in the development of neurodegenerative diseases. Disorder of gastrointestinal homeostasis leads to a shift in the composition and proportions of microorganisms inhabiting the gastrointestinal tract with subsequent production of microbial toxins, metabolites, cytokines, neurotransmitters, neuropeptides, chemokines, and endocrine messengers that impact brain functions. This bidirectional relationship also involves the release of molecules by the brain, for instance, neurotransmitters, that stimulate gut function. Ach—acetylcholine; 5-HT—5-hydroxytryptamine; IL-6—interleukin 6; TNF—tumor necrosis factor. Created with BioRender.com.

A new research direction in neurobiology and neurology aims to elucidate the role of the intestinal microbiota in the pathogenesis of NDD, in particular AD, as the basis for developing new approaches to the prevention and treatment of the pathology [199,200]. Increasing evidence suggests that the gut flora is not only involved in maintaining gastrointestinal homeostasis but may have a marked effect on other organs, including CNS, and on its disorders through the production of numerous neurotoxic substances such as lactic acid, pro-inflammatory cytokines, ammonia, and other metabolites with neuromodulatory properties. It is assumed that these substances can cause memory impairment and other cognitive disorders (Figure 6) [199,200,201].

Clinical studies have shown differences in the composition and quantity of intestinal microflora among sick and healthy people. AD patients show decreased abundance of Firmicutes and Proteobacteria and an increased abundance of Bacteroidetes [202,203]. An increase in *Shigella/Escherichia* (pro-inflammatory microbes) and a decrease in *Escherichia rectal* (anti-inflammatory) were also observed [204]. Microorganisms producing butyrate, which affects cognitive functions, have been found in AD patients [205]. Studies on sterile or broad-spectrum antibiotic-treated mice have shown that the complete absence of intestinal microflora leads to impaired brain function in learning, recognition, and behavior [206,207].

A direct relationship has been demonstrated between *Helicobacter pylori*, *Borrelia burgdorferi*, or *Chlamydia pneumoniae*, on the one hand, and elevated levels of Aβ40 and Aβ42, as well as increased release of inflammatory mediators, on the other [208,209]. In vitro studies also found that *Helicobacter pylori* indirectly activates GSK-3β and induces tau hyperphosphorylation [210]. High levels of bacterial lipopolysaccharides have been detected in the brains of AD patients [211]. Antibiotic-modified gut microflora can also influence neuroinflammation and amyloidosis in an AD mouse model [212], while healthy gut microbiota can reduce Aβ aggregates and tau pathology in the brains of AD mice [213].

Animal studies have shown that probiotic preparations containing live bacterial strains (*Lactobacillus acidophilus*, *L. plantarum*, *L. paracasei*, *L. delbrueckii* subsp. *bulgaricus*, *L. brevis*, *Bifidobacterium longum*, *B. breve*, *B. infantis*, and *Streptococcus thermophilus*) can prevent the formation of toxic Aβ aggregates and improve cognitive functions in experimental animals [214]. Similarly, probiotics improved cognitive function and reduced the number of amyloid plaques in the hippocampus of AD mice [215]. Similar results have emerged with a prebiotic based on fructooligosaccharides from *Morinda officinalis* [216]. These data indicate that normalization of intestinal biocenosis with the help of probiotic/prebiotic therapy may improve memory and cognition by reducing the levels of pro-inflammatory biomarkers and suppressing oxidative processes [217,218], opening new approaches to manage NDDs. Marine natural products have also attracted significant attention as agents to modify the gut–brain access toward neuroprotection [199].

This overview illustrates the multitude of pharmacological targets for potential NDD treatment, many of which have already been approached with marine compounds [107,108,109,144,145,148,163,164,169,199,219]. The next section turns our attention back to the Russian Pacific region, with examples of NDD-relevant marine compounds emanating from its fauna, flora, and microbiota.

## 3. Marine Natural Products from the Russian Pacific for NDD Treatment and Prevention

### 3.1. Sea Lipids from the Russian Pacific: Pharmacology and Biotechnology

#### 3.1.1. Polyunsaturated Fatty Acids (PUFAs) and Their Derivatives

Marine biotechnology, associated with the production of bioactive lipids from marine organisms, and the study of the therapeutic and prophylactic properties of these compounds is the main direction of research of the Laboratory of Comparative Biochemistry and the Laboratory of Pharmacology of the A.V. Zhirmunsky National scientific centre of marine biology (NSCMB). We will focus on this center’s most significant work in the field of lipidology.

The Laboratories studied the composition of lipids and fatty acids in the muscles and hepatopancreas of crabs inhabiting the Sea of Japan and the Sea of Okhotsk, namely the king crabs of the genus *Paralithodes* (*P. camtschaticus* and *P. platypus*, the main commercial species that are very popular in the countries of Southeast Asia), as well as of deep-sea species *Chionoecetes opilio*, *C. angulatus*, and *C. japonicus*, previously not of commercial importance but recently introduced into the crab industry. The low cholesterol content of the crab meat, the presence of all essential amino acids, and the high content of PUFAs of the ω-3 series, namely 20:5-ω-3 and 22:6-ω-3, characterize this product as an important functional food. The hepatopancreas of decapods is usually not used for food. Still, a very high proportion of triglycerides in total lipids of this organ invites us to consider it as a source of ω-3 PUFAs [220].

Of the lipids of marine origin, special attention is paid to 1-O-alkyl-glycerols (O-AKGs), a class of ester lipids formed from 1-O-alkyl-2,3-diacyl-sn-glycerols (DAGEs) by deacylation. 1-O-alkyl-sn-glycerols (AGs) are ethers of glycerol and fatty alcohols. These compounds are precursors in the biosynthesis of plasmalogens, phospholipids with an alkenyl bond at the sn-1 position of glycerol. The first stages of plasmalogen biosynthesis, namely the formation of an alkyl bond in the molecule of the dihydroxyacetone phosphate, occur in peroxisomes, and peroxisome dysfunction is one of the causes of NDD, as is the case of decreased levels of plasmenyl phosphatidylethanolamine with docosahexaenoic acid in the sn-2 position of glycerol in AD neurons [221]. It is assumed that the inclusion of AGs and DHA precursors of plasmalogens to the diet will increase their levels in brain cells; various synthetic precursors of plasmalogens [222] or semisynthetic plasmalogens [223] have been proposed for the correction of NDD, under the condition of DHA incorporation into the sn-2 position of glycerol. However, this approach is associated with complex chemical or biochemical synthesis, which may hinder practical applications. Isolation of highly purified AGs is a rather difficult task, despite the simple structure and high concentration in marine oils. The closeness of the physicochemical properties of triacylglycerols and DAGEs complicates the separation of these products. This problem can be solved by using marine organisms as a source of DAGEs.

Marine organisms accumulate large amounts of DAGEs. High concentrations of bioactive O-AKGs with a variety of beneficial effects [224] have been found in the liver of deep-sea sharks from southern Australian waters [225], in pyloric ceca, in ovaries of starfish from the Sea of Japan [226], corals [227], pteropods from deep regions of the Southern Ocean [228], and other marine organisms [229]. For the Russian Pacific inhabitants, the content and composition of O-AKGs have been determined in the hepatopancreas of the crab *Paralithodes camtschaticus* trawled in the Tatar Strait, in the liver of the squid *Berryteuthis magister* fished in the Bering Sea, and in the liver of the deep-sea stingray, *Bathyraja parmifera* fished in the Sea of Japan. Lipids from these sources are mostly (97.1–99.4%) neutral, with DAGEs present as 14.4 and 6.8% of total crab and stingray lipids, respectively. Out of O-AKGs, the main components of the crab lipids are O-AKG 16:0 and 1-O-octadecyl-sn-glycerol (AKG 18:0); O-AKG 16:0 and O-AKG 18:1 are the main components of skate liver lipids [230]. The liver of the squid *B. magister* presents the richest source of DAGEs (46.5% of total lipids), with O-AKG 16:0 prevailing [230,231]. The *B. magister* liver—making up to 20% of the weight of this commercial squid that is typically discarded—additionally contains biomedically important 1-O-alkyl-2,3-diacyl-sn-glycerols and triacylglycerols [230,232], chimyl alcohol, EPA, DHA, and stearidonic acid [233].

In the Laboratory of Pharmacology of NSCMB, the effects of dietary supplementation AGs and ω-3 PUFA have been studied in old rats. In addition to the correction of hematological parameters otherwise decreased in senile rats [234], this supplementation reduces expression of the pro-inflammatory cytokine IL-1β in the hippocampus in animals with experimental neuropathic pain, preventing activation of M1 microglia, normalizing hippocampal neurogenesis and working memory in the animals [235]. Administration of AGs in this model further prevents the development of characteristic behavioral changes and significantly increases the number of newly formed neurons in the dentate gyrus subgranular zone. Simultaneously, there has been a decrease in immunostaining of the pro-inflammatory microglial marker CD86 and a gradual predominance of the microglial phenotype M2 [235].

Marine-derived long-chain ω-3 PUFAs can influence the intensity of neuroinflammation and its cognitive consequences. Consumption of EPA and DHA has been reported to improve cognitive functions [236] and reduce the risk of dementia [237,238]. In the Laboratory of Pharmacology of NSCMB, preventive effects of a PUFA mixture (EPA + DHA) have been investigated in rats with LPS-induced neuroinflammation, revealing that PUFAs prevent the LPS-induced decline in working memory and motor activity [239]. Prophylactic administration of ω-3 PUFAs was further found to prevent the development of micro- and astrogliosis in the hippocampus with neuroinflammation while maintaining normal morphological characteristics of microglia and astrocytes. Finally, PUFAs prevented the rise in IL-1β-positive cells and in malondialdehyde (reflecting increased lipid peroxidation) in the hippocampus, overall speaking of the significant antioxidant activity of the administered compounds [239].

In the chronic neuropathic pain animal model, parenteral administration of DHA decreased the pain reaction intensity and prevented the development of neurodegenerative changes [240]. In rats with experimental spinal cord injury, DHA promoted faster and more complete recovery of the motor function of the lower extremities. Immunohistochemical mapping of myelin basic protein showed that DHA induces remyelination processes both in the center of the lesion and in the rostral and caudal segments of the spinal cord in the postoperative period [241]. These data agree with the findings in a mouse model of multiple sclerosis that ω-3 PUFA supplementation reduces cuprizone-induced demyelination and improves motor and cognitive functions [242].

In aged C57BL/6 mice with the neuropathic pain model, AGEs could prevent neuropathic pain-induced effects, including M1 microglia activation, impaired neurogenesis, and memory impairment [243]. In the same model, DHA from the *B. magister* liver has demonstrated analgesic and neuroprotective effects upon subcutaneous administration [240,244]. DHA could improve the morphological state of the sciatic nerve, prevent degradation of myelin basic protein, and stabilize the level of astroglial activity and substance P-positive nerve fibers in the superficial lamellae of the dorsal horns of the spinal cord. It also suppresses microglial activation and the synthesis of pro-apoptotic p53 in the spinal ganglia. Peripheral effects of DHA have included decreased demyelination of the constricted nerve, inhibition of astrocytosis in the dorsal horns of the spinal cord, and decreased apoptosis in spinal ganglion neurons, which together stabilized the SP-ergic afferent flow [240].

It is assumed that the neuroprotective effect of DHA in the distal segment of the damaged sciatic nerve is aimed at maintaining the morphological and functional homeostasis of Schwann cells. This effect is explained by systemic anti-inflammatory and antioxidant mechanisms of DHA in the area of peripheral nerve damage due to a decrease in the release of pro-inflammatory cytokines (IL-1β, IL-6, TNF-α) and chemokines due to the formation of a highly active metabolite, neuroprotectin D1, as well as by increasing the activity of SOD, both contributing to the reduction of neurogenic pain [241,244].

An original technology to purify N-docosahexanoylethanolamine (N-DHEA) from by-products of salmon caught in the Bering Sea was developed at the Laboratory of Comparative Biochemistry of the NSCMB [233]. To obtain ethanolamines, PUFAs were converted to ethyl esters and then treated with ethanolamine, followed by isolation of PUFA ethanolamides at the purity of 99.4%. Being a metabolite of DHA, N-DHEA is currently regarded as a potent mediator of the neurotrophic and neuroprotective effects of DHA. The history of this ethanolamine began when the effects of DHA on neurite outgrowth and synaptogenesis in cultured hippocampal neurons were studied [245,246]. DHA was found to be converted to N-DHEA in the cultures as well as in the brain and retina, with N-DHEA being >10-fold more potent than DHA in stimulating neurite outgrowth, synapse formation, and synaptic activity [247]. Due to the powerful synaptogenic activity and amide structure, the term “synaptamide” was proposed for this metabolite [248]. Synaptamide can penetrate BBB [249], promotes neurogenic differentiation of neural stem cells and neurite outgrowth, facilitates synaptogenesis in cortical neurons, and stimulates glutamatergic synaptic activity in hippocampal neurons. It attenuates LPS-induced neuroinflammatory responses in primary microglia and reduces the harmful effects of ethanol on the differentiation of neurogenic neural stem cells by suppressing TNF-α expression and NO production in challenged microglia cultures [247,250,251]. These effects of synaptamide are similar to those of DHA, but synaptamide is 1–2 orders of magnitude more potent than DHA [245,246]. Synaptamide significantly increases neurogenesis in neural stem cells already at 1 nM, increasing cAMP and activating PKA and CREB [250].

The Laboratory of Pharmacology of NSCMB has studied the effects of synaptamide on behavioral, morphological, and biochemical parameters in rats with LPS-induced neuroinflammation. The animals reveal reduced spatial working memory and long-term memory, accompanied by increased microglial activity in the CA1 region, decreased complexity of the dendritic tree of CA1 pyramidal neurons, and impaired synaptic plasticity of the hippocampus. Subcutaneous administration of synaptamide in these animals improves the morphological and biochemical parameters of the hippocampus and prevents memory impairment, restores LTP, and prevents impairments in synaptic plasticity, neuronal degeneration, and deterioration in neurogenesis, probably due to the strong anti-inflammatory activity [252].

In another study, N-DHEA was isolated from the by-products (amounting to 52% of the total weight of the animal) of the Bering Sea *B. magister* squid. In the rat chronic neuropathic pain model, squid-derived N-DHEA has prevented pain-caused sensory and behavioral changes, protected from a reduction in working and long-term memory, reduced anxiety, weakened microglia activation, suppressed release of pro-inflammatory cytokines, and reverted the decreased neurogenesis in hippocampus [253]. Long-term (5 weeks) application of the synaptamide leads to the accumulation of brain N-acylethanolamides (palmitoyl ethanolamide and oleoyl ethanolamide), apparently through N-DHEA hydrolysis. Thus, the long-term anti-inflammatory effects of N-DHEA might be mediated by an increase in palmitoyl ethanolamide in the brain, which is known to reduce neuroinflammation [254].

In rats with mild traumatic brain injury, N-DHEA from *B. magister* was administered subcutaneously to help restore cognitive functions impaired as a result of trauma, reduce anxiety, and decrease pro-inflammatory markers of microglia. In vitro, N-DHEA was found to suppress LPS-induced neuroinflammation by inhibiting the production of ROS, NO, nitrite, IL-1β, and CD86, as well as by stimulating SOD synthesis [255]. Comparative in vitro and in vivo evaluation of the anti-inflammatory activity of N-DHEA (synaptamide) and N-eicosapentaenoylethanolamine (N-EPEA) obtained from by-products of salmon caught in the Bering Sea [256] has shown that both compounds prevent LPS-mediated production of pro-inflammatory cytokines TNF-α and IL-6 in SIM-A9 mouse microglia culture. In mice, synaptamide, unlike N-EPEA, reverses LPS-induced hippocampal TNF-α and IL-1β. At the same time, both compounds promote the M2 phenotype in microglia and prevent LPS-mediated astrogliosis [257]. Despite the more pronounced anti-inflammatory activity of synaptamide, both N-DHEA and N-EPEA are effective in maintaining normal levels of hippocampal LTP in neuroinflammation. Both compounds thus emerge as having high therapeutic potential.

Another promising source of marine lipids for NDD prevention and treatment is Pacific saury (*Cololabis saira*), fish of the Scomberesocidae family endemic to the Northern Pacific Ocean, where it is widely distributed from Asian coasts to North America. It is one of the most important fish species in the region, commercially caught by Japan, Russia, Taiwan, Korea, and the Democratic People’s Republic of Korea. The annual catch of saury can exceed 500 thousand tons. The detailed analysis demonstrates that with the overall annual catch of up to 860 thousand tons, the natural replenishment of the saury will not be compromised. The Russian saury fishery dates back to 1958; the maximum catch of 110 thousand tons was achieved in 2007. The total commercial stock of Pacific saury in the South Kuril fishing area is at least 2 million tons. Constituting >10% of the fish mass, the entrails of Pacific saury contain 25% crude fat rich in phospholipids, up to 7.6% [258] such as phosphatidylcholine, phosphatidylinositol, and phosphatidylethanolamine, also including significant amounts of PUFAs [259]. Saury phospholipids were found to strongly suppress Aβ42 secretion from CHO cells stably transfected with APP and presenilin, suggesting that Pacific saury visceral phospholipids rich in ω-3 fatty acids, especially DHA, have the potential to correct NDD [259]. Numerous studies reviewed above prove the ability of DHA to enhance brain development, cognition, and learning. Readily passing across BBB, DHA demonstrates neuroprotection in mouse and rat models of AD [260,261]. Further, most clinical studies indicate the preventive effect of DHA on AD progression [262,263,264,265]. These data open the possibility of industrial isolation of health-promoting compounds from Pacific saury, primarily of NDD preventive supplements.

#### 3.1.2. Sphingolipids

Sphingolipids (SLs) were discovered as structural components of brain cell membranes in 1874, and it is now clear that their composition and metabolism are important for brain development and synaptic plasticity [266]. Mutations altering SL metabolism lead to abnormal SL deposition causing severe cognitive retardation, and imbalance of SLs contributes to neurological disorders including AD, PD, or depression [267,268]. SLs are based on the aliphatic amino alcohol sphingosine and include sphingomyelins, cerebrosides, and gangliosides. Sphingomyelins contain a polar head, which includes phosphatidylcholine or phosphoethanolamine residues, so the sphingomyelin molecule carries both positive (choline residue) and negative (phosphoric acid residue) charges, as well as two non-polar tails: a long aliphatic chain of sphingosine and an acyl radical of a fatty acid. Cerebrosides (aka glycosphingolipids) do not contain phosphoric acid residues. Therefore their polar heads do not carry a charge but contain one or more carbohydrate residues, for example, galactose (galactocerebrosides of brain neuron membranes) or glucose (glucocerebrosides of membranes of other cells), as well as N-acetyl-D-galactosamine. Glycosphingolipids containing oligosaccharides are called globosides or gangliosides (with one or more sialic acids linked to the sugar chain) [269]. Glucosylsphingolipids are mainly formed by ceramide, considered the central product of SL metabolism and formed either by the breakdown of sphingomyelin (the catabolic pathway) or by the anabolic pathway known as SL synthesis. Ceramides are composed of a sphingosine backbone and a fatty acid residue. There exists the so-called ceramide paradox: ceramide can be harmful by causing cell death but also beneficial by arresting neural progenitor cell cycle and promoting neurite outgrowth [270].

It is now clear that SLs and their metabolites play an important role in several cellular processes and signaling events, including neuroinflammation. The main signaling molecules of SLs, namely ceramide and sphingosine-1-phosphate (S1P), can initiate both pro- and anti-inflammatory responses by reacting to the activation of their modulators, sphingomyelinases and sphingosine kinases. It is believed that short-chain ceramides (acyl chain length C2-C8) exhibit an anti-inflammatory effect, while long-chain ceramides (C16-C24) initiate a pro-inflammatory response [271]. Sphingosine-1-phosphate (S1P), formed upon phosphorylation of sphingosine by sphingosine kinase activation, plays an important role in intracellular and extracellular signaling in the CNS, being involved in migration, proliferation, and changes in astrocyte and microglia morphology, thereby participating in neuroinflammation. Binding to receptors, S1P induces the proliferation of target cells and the synthesis of pro-inflammatory cytokines and neurotoxic molecules (ROS and NO). Accumulating in the extracellular space, S1P activates microglia and further enhances the inflammatory response [272]. By blocking S1P receptors with antagonists, pro-inflammatory responses can be reduced [273]. Overall, ceramide metabolism may be a therapeutic target to prevent and reverse neuroinflammation in neurodegenerative pathology.

Out of inhabitants of the Russian Pacific, 146 SLs with an exceptionally large variety of structural types of ceramides, cerebrosides, and gangliosides have been isolated from representatives of the Echinodermata phylum, including 15 species of starfish (class Asteroidea) and nine species of sea cucumbers (class Holothuroidea) [274]. These findings illustrate that echinoderms are a rich source of SLs, with structures often markedly different from those of the corresponding plant and terrestrial animal metabolites. A number of the echinoderm SLs revealed potent neuritogenic activity, as observed for:

- 1-O-[(N-acetyl-α-D-neuramynosyl)-(2→8)-(N-acetyl-α-D-neuraminosyl)-(2→3)-β-D-galactopyranosyl-(1→4)-β-D-glucopyranosyl]-ceramide from the starfish *Luidia maculata* [275];
- 1-O-α-L-arabinofuranosyl-(1→3)-α-D-galactopyranosyl-(1→4)-(N-acetyl-α-D-neuraminosyl)-(2→6)-β-D-galactofuranosyl-(1→3)-[α-L-arabinofuranosyl-(1→4)]-α-D-galactopyranosyl-(1→4)-(N-acetyl-α-D-neuraminosyl)-(2→3)-β-D-galactopyranosyl-(1→4)-β-D-glucopyranoside of ceramide composed of heterogeneous (2S,3S,4R)-phytosphingosine (iso-C-17-phytosphingosine as the major component) and (2R)-2-hydroxy fatty acid units (docosanoic acid as the major component) from the starfish *Patiria* (=*Asterina*) *pectinifera* [276];
- 8-O-methyl-(N-glycolyl-α-D-neuraminosyl)-(2→11)-(N-glycolyl-α-D-neuraminosyl)-(2→11)-(N-glycolyl-α-D-neuraminosyl)-(2→3)-β-D-galactopyranosyl-(1→4)-β-D-glucopyranoside of a ceramide composed of phytosphingosines and 2-hydroxy n-fatty acids from the starfish *Linckia laevigata* [277];
- α-NeuAc-(2→4)-α-NeuAc-(2→3)-β-Gal-(1→8)-α-NeuAc-(2→3)-β-GalNAc- (1→3)-β-Gal-(1→4)-β-Glc-(1→1)-Cer from the sea cucumber *Apostichopus* (=*Stichopus*) *japonicus* [278];
- three gangliosides from the sea cucumber *Stichopus chloronotus* [279];
- eight gangliosides from the sea cucumber *Pseudocnus echinatus* (=*Cucumaria echinate*) [280,281].

These findings highlight the pharmacological potential of SLs from North Pacific invertebrates for applications in NDDs.

### 3.2. Sea Sterols and Oxysterols

Marine sterols are structurally and functionally similar to cholesterol. Due to this structural similarity and the use of the same absorption pathways, marine sterols (and dietary sterols in general) are able to reduce cholesterol absorption in the intestine and thus participate in the maintenance of cholesterol homeostasis, the disturbance of which contributes to the pathobiology of various neurological diseases. In addition to lowering cholesterol levels, dietary sterols have a protective effect against apoptosis, oxidative stress, and neuroinflammation by modulating cell survival signaling systems such as BDNF, nuclear factor-erythroid 2-related factor 2 (Nrf2), and NF-κB signaling [282]. In silico modeling of absorption, distribution, metabolism, excretion, and toxicity (ADME/T) as a rational drug design tool has been applied to analyze the potential of marine sterols as drug candidates. Fucosterol, the most abundant seaweed sterol, conforms to Lipinski’s rule of five and Jorgensen’s rule of three, indicating its drug-likeliness. In addition, fucosterol is likely to penetrate BBB [283].

Marine sterols, including fucosterol and saringosterol, have been proposed to act as anti-AD agents, affecting oxidative stress, inflammation, cholinergic deficiency, amyloidogenesis, the cholesterol homeostatic pathway, and signaling pathways associated with neuronal survival. Fucosterol and two other sterols, 3,6,17-trihydroxy-stigmasta-4,7,24(28)-triene and 14,15,18,20-diepoxyturbinarin, isolated from the brown algae *Silvetia* (=*Pelvetia*) *siliquosa*, protected against CCl_4_-induced oxidative stress in rats by increasing the levels of SOD, GPX1, and CAT [284]. Fucosterol isolated from edible brown algae *Eisenia bicyclis* inhibited ROS production in RAW264.7 macrophages treated with tert-butyl hydroperoxide (t-BHP) [285]. In HepG2 cells treated with t-BHP and tacrine, fucosterol attenuated oxidative stress, causing a decrease in ROS and an increase in glutathione levels [286]. Fucosterol from *Sargassum aquifolium* (=*S. binderi*) protected against oxidative stress in a particulate-treated A549 human lung epithelial cell injury and inflammation model by accumulating SOD, CAT, and heme oxygenase 1 (HO-1) in the cytosol and Nrf2 in the nucleus [287]. 7-Dehydroerectasteroid F isolated from the soft coral *Dendronephthya gigantea* protected PC12 cells from H_2_O_2_-induced oxidative damage by enhancing Nrf2 nuclear translocation and subsequent activation of HO-1 expression [288].

When exposed to toxic stimuli, microglia increase expression of inducible NO synthase (iNOS) and cyclooxygenase-2 (COX-2) and secrete inflammatory mediators such as TNF-α, IL-6, and IL-1β that can cause neuronal degeneration. Fucosterol reduced secretion of IL-1β, IL-6, TNF-α, NO, and PGE2 in LPS- or Aβ-treated microglial cells, thereby suppressing inflammation [289]. It also suppressed the expression of COX-2 and iNOS and the NF-κB signaling in RAW 264.7 macrophages stimulated with LPS [286]. Fucosterol has been able to attenuate LPS-mediated inflammation by suppressing NF-κB activation and stimulating alveolar macrophages in mice [290]. In particulate-treated human lung epithelial cells, fucosterol from *S. aquifolium* inhibited NF-κB activation and nuclear translocation and phosphorylation of MAPK, ERK1/2, JNK, and COX-2 [287]. In LPS-treated activated mouse macrophages RAW264.7, fucosterol from *Undaria pinnatifida* suppressed expression of iNOS, TNF-α, and IL-6, inhibited NF-κB activation and nuclear translocation, and attenuated activation of MAPK kinases 3/6 (MKK3/6) and MAPK-activated protein kinase 2 [291]. Two steroids, 5α-pregn-20-en-3β-ol, and 5α-cholestan-3,6-dione, isolated from the octocoral *Dendronephthya mucronate*, inhibited LPS-induced NO production in RAW264.7 cells [292]. Another sterol, dendronesterone D from *Dendronephthya* sp., inhibited iNOS and COX-2 expression [293]. Marine sterols have also been shown to inhibit cholinesterase activity. Fucosterol and 24-hydroperoxy 24-vinylcholesterol inhibited AChE and BChE [289,294], acting as non-competitive inhibitors [295].

Marine sterols may also have an anti-amyloidogenic potential. Thus, fucosterol can act as a non-competitive [296] and an active site-binding inhibitor of BACE1 [297]. The neuroprotective effect of marine sterols is manifested in the suppression of Aβ aggregation and neuroinflammation. Fucosterol has been shown to protect against Aβ42-mediated cytotoxicity by upregulating tropomyosin receptor kinase B (TrkB)-mediated ERK1/2 signaling and enhancing BDNF expression in the dentate gyrus [298]. Fucosterol pretreatment attenuated Aβ-induced neurotoxicity in SH-SY5Y cells by upregulating neuroglobin mRNA expression and also decreased APP mRNA and Aβ levels [299]. 24(S)-saringosterol from *Sargassum fusiforme* increased ApoE secretion in astrocytes, and 24(S)-saringosterol-treated astrocyte-conditioned medium increased microglial clearance of Aβ42. 24(S)-saringosterol also reduced Aβ42 release in N2a neuronal cells overexpressing APP [300]. 16-O-desmethylasporyergosterol-β-D-mannoside from the marine fungus *Dichotomomyces cejpii* showed moderate activity in reducing Aβ42 in N2a cells [301]. 4-Methylenecholestane-3β,5α,6β,19-tetraol attenuated glutamate-induced neuronal damage, prevented NMDA-induced increase in intracellular calcium, and inhibited NMDA currents, thus demonstrating the therapeutic potential of sterols in relation to glutamate excitotoxicity [302].

Activation of the nuclear receptor LXR-β (liver X receptor-beta) induces several reverse cholesterol transport genes, including ApoE, ATP-binding cassette transporter (ABCA1), ATP binding cassette subfamily G member 1 (ABCG1), and sterol regulatory element-binding protein 1 (SREBP1). This nuclear receptor plays an important role in protection against neurodegeneration [303]. After ligand activation, LXR-β compensates for the dopaminergic loss [304], reduces the toxic load of mutant huntingtin [305], and also accelerates Aβ clearance [306]. Fucosterol, being a selective LXR-β agonist, increased the expression of LXR-β target genes encoding ABCA1, ABCG1, and ApoE [307], promoting cholesterol homeostasis in the brain and Aβ clearance. Saringosterol, also a selective LXR-β agonist, stimulated the transcription of ABCA1, ABCG1, and SREBP-1c and is considered a natural hypocholesterolemic agent [308].

Thus, marine sterols are neuroprotective by attenuating pathobiological processes such as oxidative stress, inflammation, Aβ42-induced apoptosis, and perturbation in cholesterol homeostasis.

Oxysterols or cholesterol-oxidized products (COPs) are formed by spontaneous and/or enzymatic oxidation of cholesterol on the steroid core or side chain. Unlike cholesterol, some COPs can easily cross BBB leading to increased inflammation that damages brain cells. The ability of COPs to induce severe dysfunction in organelles (especially mitochondria) plays a critical role in redox homeostasis, inflammation, lipid metabolism, and the regulation of cell death [309].

The involvement in the pathogenesis of PD and AD has been established for two brain oxysterols, 24S-hydroxycholesterol and 27-hydroxycholesterol [23,310]. Other oxysterols such as 7β-hydroxycholesterol, 7-ketocholesterol, 3β,5α-dihydroxycholestan-6-one, 7α-hydroxy-3-oxocholest-4-enoic acid, 7α,25-dihydroxycholest-4-en-3-one, and (25R)-7α,26-dihydroxycholest-4-en-3-one, were found to be exported from the brain and could also be implicated in NDD [311]. Thus, unusual oxysterols of marine origin become interesting in the context of their potential biological effects in NDD. It has recently been shown that hecogenin and cholest-4-en-3-one isolated from the marine worm *Urechis unicinctus* can inhibit BACE1 [312]. In contrast, 24-methylene-5-cholestene-3β,7α-diol from the finger sponge *Haliclona oculata* strongly inhibited BChE [313].

Fourteen individual oxysterols, including four novel compounds, have been isolated from the marine sponge *Piloderma* sp. (=*Inflatella* sp.) of the Sea of Okhotsk and tested in a PD cell model using Neuro2a cells treated with 6-hydroxydopamine. Of all the oxysterols studied, two new compounds, (22E)-24-nor-cholesta-5,22-diene-3β,7α-diol and (22E)-24-nor-cholesta-5,22-diene-3β,7β-diol, as well as one known compound (22E,24R)-24-methylcholesta-5,22-diene-3β,7α-diol, significantly increased cell survival and reduced ROS production to normal values; (22E)-24-nor-cholesta-5,22-diene-3β, 7β-diol was the strongest and became a candidate for further research [314].

### 3.3. Bioactive Compounds of Marine Algae

Marine macroalgae, more commonly known as seaweeds, include the class of green algae (Chlorophyceae, phylum Chlorophyta), the class of brown algae (Phaeophyceae, phylum Ochrophyta), and the phylum of red algae (Rhodophyta). According to N.G. Klochkova, 73 species of green algae, 159 species of brown algae, and 319 species of red algae inhabit the Far Eastern seas [315].

In the Russian Pacific, brown algae form algal belts along the entire Far East coast. The most valuable are macrophytes belonging to two orders: Laminariales (kelp) and Fucales (fucus). Their reserves are estimated at 25–28 million tons. In Russia, 19 algal species are certified for commercial harvesting, but only a few are massively collected. The current understanding of the resource potential of the Far Eastern marine macrophytes is limited to the fishing areas. The most productive thickets of commercial brown algae are in the southern Kuril Islands, where the total biomass of commercial species is estimated at 1065 thousand tons, in the Lesser Kuril Ridge (924.3 thousand tons), on the eastern coast of Kamchatka (300–350 thousand tons), on the southeastern coast of Kamchatka (180 thousand tons), in the northern Kuril Islands (256 thousand tons), in the Shantar Islands (370–420 thousand tons), in the southeastern part of Sakhalin Island (694 thousand tons). In the Sea of Japan in the Primorye coastal region, the total biomass of commercial and promising macrophytes is estimated at 130–150 thousand tons. In other areas of the Far East coast, algae stocks are dispersed over much larger areas, and there are no data on their total biomass. However, based on the studies, the total stock of brown algae in the fishing areas is estimated at >3 million tons [316].

The kelp algae are most productive in the Bering Sea. In the Gulf of Anadyr alone, the reserves of *Saccharina* (=*Laminaria*) *gurjanovae* are estimated at 200 thousand tons. The Sea of Okhotsk is dominated by *S. gurjanovae*, *Alaria marginata*, and *Stephanocystis* (=*Cystoseira*) *crassipes*. On the coast of Kamchatka, the main commercial species is *Hedophyllum* (=*Laminaria*) *bongardiana*. The total reserves of this algal species alone in the Far Eastern seas are estimated at 500–700 thousand tons. In the northern Kuril Islands, the most significant are the stocks of *Arthrothamnus bifidus* and *Eualaria* (=*Alaria*) *fistulosa*. The reserves of the latter in the coastal waters of the northern and middle Kuril Islands amount to 2.5–3 million tons. In the coastal zones of the Magadan Region and the Khabarovsk Territory, the reserves of *S. crassipes* amount to 7.5 million tons. The reserves of the fucus algae *Fucus distichus* subsp. *evanescens* (=*Fucus evanescens*), which grows in all the Far Eastern seas, reach 500–700 thousand tons. From the southern Kuril Islands to the south of Primorye, the main commercial species is *Saccharina japonica*, the stock of which only in the coastal zone of the southern Kuril Islands exceeds 400 thousand tons. Other commercial algae are dominated by *Saccharina* (=*Laminaria) angustata* and *Saccharina kurilensis* (*Cymathaere japonica*). *Arthrothamnus kurilensis*, *E. fistulosa*, and *A. marginata* are considered promising macrophytes for commercial purposes. In the coastal waters of Sakhalin, large stocks of *S. japonica* have been identified near the southwestern and southeastern extremities of the island. In the Sea of Japan, *S. japonica* is also the main commercial species. In the northern part of the Tatar Strait, near the mainland coast, on a 500-km stretch from Cape Zolotoe to Cape Yuzhny, the stocks of *S. japonica*, *S. sculerpa*, and *Alaria ochotensis* have been estimated at 65.5 thousand tons. The mass species of the coast of Primorye are *S. japonica* (65.0 thousand tons), *Costaria costata* (14.8 thousand tons), and *S. crassipes* (1.0 thousand tons) [316].

The Far Eastern marine flora of red algae includes more than 250 species, of which many are of interest for harvesting, but only *Ahnfeltia tobuchiensis* is currently harvested at the commercial scale. The main resource base of *A. tobuchiensis* is the Far Eastern fishery basin, particularly the Peter the Great Gulf, the Busse lagoon (Sakhalin Island), and Treason Bay (Kunashir Island). The total biomass of *A. tobuchiensis* in the Sakhalin-Kuril region, according to 2009 data, is 200.3 thousand tons. At the same time, the commercial stock totals 89.2 thousand tons, of which 98% is concentrated in Treason Bay. *A. tobuchiensis* is considered an important source of natural agar and its derivatives. The permitted catch of ahnfeltia only in the South Kuril zone is about 9 thousand tons, and in the entire basin, the recommended catch of ahnfeltia in 2013–2017 ranged from 10.7 to 11.3 thousand tons per year. Of the other red algae, the most common species that can be the object of local harvesting are *Turnerella mertensiana*, *Constantinea rosa-marina*, *Odonthalia corymbifera*, *Callophyllis rhynchocarpa*, *Callophyllis flabellata*, *Congregatocarpus kurilensis* (=*C. pacificus*), *Neohypophyllum middendorfii*, *Ptilota* (=*Neoptilota*) *asplenioides*, *Ptilota filicina*, and *Wildemania* (=*Porphyra*) *variegata* [317].

Regarding bioactive compounds, brown algae are primarily the source of phlorotannins, which are polymeric derivatives of phloroglucinol (1,3,5-trihydroxybenzene) with a mass of 126 Da to 1 × 10^5^ Da or more [318]. Polymer molecules of phlorotannins are formed by connecting phloroglucinine residues through C-C and/or C-O-C bonds. Depending on the bond type, phlorotannins are divided into four classes: (i) fugalols and phloretols characterized by an ether bond; (ii) fucols with a phenyl bond; (iii) fucophlorethols that have ether and phenyl bonds; and (iv) ecols and carmalols characterized by a dibenzodioxine bond [319]. Compounds within each class may be grouped as linear phlorotannins if the C-C and/or C-O-C units have only two terminal phloroglucinol residues or branched if they are linked to three or more monomers. Fucophloretols are formed by combinations of C-C and C-O-C bonds between monomeric residues leading to a large structural diversity of compounds with linear, branched, and heterocyclic variants. Heterocyclic fucophlorethols may contain dibenzodioxin and furan rings in their structure. One species of algae can contain phlorotannins with different structures and different degrees of polymerization.

Of the biological effects of phlorotannins, antioxidant and anticholinesterase activities can be mentioned. Ecol, florofucofuroecol A, diecol, and 8,8′-biecol have been shown to inhibit phospholipid peroxidation induced by superoxide and 2,2-diphenyl 1-picrylhydrazyl radicals in vitro [320]. Six phlorotannins—eckstolonol, eckol, phlorofucofuroeckol-A, dieckol, 2-phloroeckol, and 7-phloroeckol isolated from *Ecklonia stolonifera*—showed various AChE inhibitory activities; eckstolonol and phlorofucofuroeckol-A also inhibited BChE [294]. 6,6′-Biecol from *Ishige okamurae* also inhibited AChE [321]. Some terrestrial plant tannins have recently been shown to inhibit the oncogenic Wnt signaling pathway by specific inactivation of the Wnt growth factors [322]. Given the multifaceted involvement of the Wnt pathway in NDD [323], a perspective investigation could be devoted to the assessment of phlorotannins’ effects on this signaling mechanism.

The content of polyphenolic compounds (including phlorotannins) has been determined in marine brown algae belonging to the families Laminariaceae, Alariaceae, Arthrothamnaceae, Costariaceae, Cystoseiraceae, and Fucaceae, collected in the Sea of Okhotsk (Aniva and Terpeniya coves), the Pacific coast of Kamchatka (Avacha and Spaseniya coves) and in the Sea of Japan (Tatar Strait). The highest polyphenol content has emerged from *Agarum turneri*, *Thalassiophyllum clathrus*, *F. evanescens*, and *S. crassipes*. Extracts of all algae had antioxidant effects. However, the maximum activity has emerged for the alcohol and aqueous extracts of *A. turneri*. The authors consider *A. turneri*, *F. evanescens*, *T. clathrus*, *S. crassipes*, and *C. costata* as promising sources of polyphenols with high antioxidant activity [324].

Macroalgae are also sources of such valuable bioactive compounds as laminaran (or laminarin), fucoidan, fucoxanthin, alginic acid, and its derivatives, as discussed below.

#### 3.3.1. Laminarans

Laminarans are water-soluble polysaccharides (1,3;1,6-β-D-glucans) of brown algae and can constitute up to 20% of the dry algal weight. Laminarans are polysaccharides built from β-D-glucose residues linked by 1,3- and 1,6-glycosidic bonds. Their molecules can contain a mannitol (M-chain) or glucose (G-chain) residue at the reducing ends in various ratios. The 1.3/1.6 bond ratios and the types of inclusion of these bonds in laminaran molecules vary depending on the species of algae. The molecular weights of laminarans are small and usually range from 3 to 10 kDa, sometimes up to 50 kDa. Due to their relatively simple structure, low toxicity, and biological activities (antiviral, immunocorrective, antiproliferative, antimetastatic, proapoptotic, radioprotective, and radiosensitizing), laminarans and their modified derivatives are considered potential pharmaceutical substances [325].

Laminaran from the brown seaweed *Laminaria digitata* has demonstrated the capacity to induce cell cycle arrest and to inhibit heregulin-stimulated ErbB2 phosphorylation, which activates the JNK to regulate a number of processes involved in oncogenesis and neurodegenerative disorders [326]. In another study, intraperitoneal pretreatment of gerbils with *L. digitata*-derived laminaran was found neuroprotective in a model of transient global forebrain ischemia/reperfusion [327]. In another study involving aged gerbils, laminaran pretreatment effectively protected pyramidal neurons in the hippocampal CA1 field from ischemia/reperfusion injury. Laminaran significantly reduced ischemia-induced production of superoxide anions, 4-hydroxy-2-nonenal levels, and pro-inflammatory cytokine (IL-1β and TNF-α) expression in CA1 pyramidal neurons. In addition, laminaran significantly increased the expression of SOD and anti-inflammatory cytokines (IL-4 and IL-13) in CA1 neurons both before and after injury [328]. These data indicate that laminarin is able to protect neurons from ischemic brain injury by attenuating ischemia-induced oxidative stress and neuroinflammation.

#### 3.3.2. Fucoidans

Fucoidans are sulfated brown alga polysaccharides containing a backbone of alternating 1,3- and 1,4-linked α-L-fucose residues as the main component. Fucoidans can also contain galactose, mannose, xylose, rhamnose, glucose, and glucuronic acid as minor components. At least five structural groups of fucoidans can be identified based on their monosaccharide composition, and in the case of fucans—according to the type of the main chain: fucoidan molecules consisting of (i) fucose residues (1,3-α-L-fucans), (ii) fucose and sulfate (1,3; 1,4-α-L-fucanes), (iii) fucose, galactose and sulfate residues (galactofucans and fucogalactans), (iv) fucose, mannose, uronic acid residues and sulfate (fucoglucuronomannans and glucuronofucans/fucoglucuronans), and (v) fucose, galactose, mannose, xylose, glucose, arabinose, uronic acid residues and sulfate (complex heteropolysaccharides) [329]. Fucoidans are often highly branched; some further contain acetyl groups [329]. Structural characterization of fucoidans from brown algae *Saccharina cichorioides* (formerly *Laminaria cichorioides*, order/family Laminariales/Laminariaceae), *U. pinnatifida* (Laminariales/Alariaceae), which were collected from the natural habitats of Trinity Bay (the Sea of Japan), and *F. evanescens* (Fucales/Fucaceae) collected at the Kunashir Island (Pacific Coast) revealed that fucoidan from *S. cichorioides* consists of (1→3)-α-L-fucose residues [330], fucoidan from F. evanescens—of alternating (1→3)- and (1→4)-α-L-fucose [331], while fucoidan from *U. pinnatifida* is built up of (1→3)- and (1→4)-linked α-L-fucose and β-D-galactose residues [332].

Fucoidans have a variety of biological and pharmacological activities, including antitumor, antiviral, antibacterial, anti-inflammatory, radioprotective, etc., and are practically non-toxic [333,334]. The biologically active additive Fukolam^®^ based on fucoidans was developed at the PIBOC center in Vladivostok. It should be emphasized that the structural diversity of fucoidans, often dependent on the type of algae, the place and season of harvest, as well as extraction methods, significantly complicates their standardization and, accordingly, the creation of drugs.

Fucoidan isolated from the brown alga *Ecklonia maxima* suppressed BACE1 [335], while that isolated from *U. pinnatifida* and *Fucus vesiculosus* had an inhibitory effect on Aβ aggregation and attenuated Aβ42-induced cytotoxicity in PC12 cells [336]. The same fucoidan also had a neuroprotective effect in mice with d-Gal-induced cognitive dysfunction [337]. Fucoidan also attenuated Aβ-induced toxicity in a transgenic *Caenorhabditis elegans* AD model, reducing Aβ accumulation by stimulating proteolysis and suppressing ROS production [338]. Moreover, in mice with trimethyltin-induced cognitive dysfunction, a fucoidan/polyphenol extract from the kelp *Ecklonia cava* improved learning and memory, suppressing tau hyperphosphorylation and β-amyloid production [339].

Fucoidan from *S. japonica* showed a neuroprotective effect in C57BL/6 mice with a 1-methyl-4-phenyl-1,2,3,6-tetrahydropyridine-induced PD model. The treatment with fucoidan significantly corrected the motor impairment, counteracted dopamine depletion of the striatum, and promoted the recovery of tyrosine hydroxylases-positive neurons in substantia nigra pars compacta [340]. In an in vitro model of PD, fucoidan pretreatment preserved cell morphology, increased mitochondrial activity, and reduced 1-methyl-4-phenylpyridinium-induced lactate dehydrogenase release, possibly due to the prevention of ROS generation [340]. Fucoidan from *F. vesiculosus* protected rat cholinergic neurons from Aβ-induced neurotoxicity and blocked activation of caspase-9 and caspase-3 [341]. Fucoidans from the brown algae *Laminaria hyperborea* and *Saccharina latissima* have been proposed as potential therapeutics for age-related macular degeneration and other pathologies that involve lipid dysregulation, inflammation, oxidative stress, and pro-angiogenic signaling [342].

#### 3.3.3. Fucoxanthin

This natural carotenoid, a tetraterpene, is abundant in brown algae and displays multiple biological activities. It contains unique functional groups, including an unusual allenic bond and a 5,6-monoepoxide structure. Fucoxanthin isolated from the brown alga *Sargassum horneri* could reverse scopolamine-induced impairments of cognition in mice [343]. It also increased ChAT activity, BDNF expression, and noncompetitively inhibited AChE activity in the hippocampus [343]. BACE1 inhibition [296] and attenuation of secretion of pro-inflammatory cytokines, reduction in ROS generation, and increases in levels of antioxidant enzymes in Aβ-treated microglial cells [344] have also been induced by fucoxanthins.

In another study, fucoxanthin extracted from *S. horneri* inhibited the formation of Aβ42 fibrils, rendering the Aβ42 oligomers less toxic to SH-SY5Y cells. Analysis of the interactions of fucoxanthin with the Aβ42 peptide showed that nine fucoxanthin molecules grouped to bind to Aβ and form stable aggregates that inhibit the Aβ42 conformational transition and subsequent aggregation. Analysis of atomic contacts between fucoxanthin molecules and Aβ42 monomers showed that fucoxanthin directly binds to the Aβ peptide mainly through hydrophobic interactions [345].

To date, several mechanisms of anti-AD and anti-PD effects of fucoxanthin have been established: decreased formation of Aβ42 fibril and Aβ42 oligomers; inhibition of Aβ aggregation; prevention of DNA damage by ROS through increased glutathione and SOD activity; counteracting microglial activation via the Nrf2/HO-1 pathway; increased cell survival through the PKA/CREB pathway and increasing BDNF secretion; reduction of pro-inflammatory mediators (TNF-α, IL-6, IL-1β, and PGE2); decreased expression of iNOS and COX-2 and decreased MAPK pathway activation, leading to suppression of the Akt/NF-κB and MAPKs/AP-1 pathways; activation of PI3K/Akt concurrently with stopping the ERK pathway [219,344,345,346,347].

### 3.4. Echinochrome

Echinochrome A (6-ethyl-2,3,5,7,8-pentahydroxy-1,4-naphthoquinone, EchA) is a dark red pigment isolated from shells and spines of sea urchins, which together with other structurally related compounds belongs to the group of spinochrome pigments, first identified in sea urchins [348]. In the southern waters of the Sea of Japan, the main source of EchA is the sea urchin *Scaphechinus mirabilis*. EchA contains hydroxyl groups that render the molecule with antioxidant properties [349]. This powerful natural antioxidant has demonstrated curative effects in multiple disease models linked with oxidative stress and inflammation [350,351,352,353,354]. EchA is the active ingredient of a medicinal drug “histochrome”, developed at the PIBOC center, Vladivostok, and registered in Russia as a cardioprotective drug [355]. Histochrom^®^ proved to be effective in an experimental hemorrhagic stroke model, attenuating neurological symptoms and reducing ischemic cerebral edema in the affected hemisphere, which is caused by the release of free iron and oxidative stress [355].

In the experimental model of cerebral ischemic stroke in rats, EchA perfusion through the external carotid artery restored the damaged area of the brain and improved motor activity. EchA was found to neutralize iron cations accumulating in the ischemic area, to increase Bcl-2 expression while reducing the levels of caspase-3 and Bax, and to increase the levels of p-ERK/ERK, p-AKT/AKT, and BDNF [356]. In vitro, EchA was found to inhibit AChE and showed NO-scavenging activity in A7r5 (rat aortic vascular smooth muscle) cells exposed to sodium nitroprusside [357]. Structural synthetic EchA analogs, such as trimethyl ether of echinochrome A, anhydroethylidene-6,6′-bis(2,3,7-trihydroxynaphthazarin), spinochrome B, spinochrome E, echinamine A, 6-pentyl-2,3,5,7,8- pentahydroxy-1,4-naphthoquinone, 2,3,5,7,8-pentahydroxy-6-(5′-hydroxypentyl)-1,4-naphthoquinone), and dimeric spinochrome (binaphthoquinone), showed increased antioxidant activity and stimulated ATP production in AC16 human cardiomyocyte cells [358]. These results suggest that EchA and/or its structural analogs could have a general cytoprotective therapeutic potential, including the potential for neuroprotection.

### 3.5. Asterosaponines

Astrosaponins are starfish-derived steroid oligoglycosides with diverse biological activities [359]. At least 17 unique asterosaponins have been isolated, and their structures elucidated, from various starfish species of the Russian Pacific. These include hippasteriosides A-D from *Hippasteria phrygiana* (=*H. kurilensis*) [360] or laevigatoside from *L. laevigata* [361], both collected at Matua islands (Sea of Okhotsk); aphelasteroside F from *Aphelasterias japonica* (Posyet Bay, Sea of Japan) [362]; leptasteriosides A-F from *Leptasterias ochotensis* (Bolshoy Shantar island, Sea of Okhotsk) [363]; hylodoside A from *L. hylodes reticulata* (Sea of Okhotsk) [364], lethasteriosides A and B from *Lethasterias fusca* (Posyet Bay, Sea of Japan) [365], cheliferoside L1 from *Lethasterias nanimensis chelifera* (Shiashkotan island, Kuril Islands) [366], and aphelasteroside C from *A. japonica* (Posyet Bay, Sea of Japan) [367]. All of these asterosaponins have been attributed to anti-inflammatory properties [359].

More astrosaponins have been isolated from the starfish *P. pectinifera* (asterosaponin P1, (25S)-5α-cholestane-3β,4β,6α,7α,8,15α,16β,26-octaol, and (25S)-5α-cholestane-3β,6α,7α,8, 15α,16β,26-heptaol) and *Distolasterias nipon* (distolasterosides D1-3). These polar steroids enhanced neurite outgrowths in C-1300 mouse neuroblastoma cells in nanomolar concentrations and showed synergistic effects with NGF and BDNF [368,369,370]. In oxygen-glucose deprivation experiments, these compounds demonstrated neuroprotective effects in C-1300 cells and organotypic cultures of hippocampal slices [370]. Neuritogenic activities in PC12 pheochromocytoma cells have also been found for astrosaponins from *L. laevigata* collected in the Sea of Japan [371]. In this regard, it should be emphasized that endogenous neurotrophins NGF and BDNF, providing a promise in the potential treatment of damaged neurons, have limitations in medical applications due to their large mass and difficulty in passing BBB [372]. Therefore, low molecular weight compounds that mimic the activity of neurotrophins (or boost the activity of the locally available factors) and are able to cross the BBB are of therapeutic interest for the treatment of traumatic or ischemic brain injuries and neurodegenerative diseases [370].

### 3.6. Marine Alkaloids

Manzamines and manzamine-related compounds are a group of polycyclic marine sponge alkaloids distinguished from other alkaloids by a previously unknown skeletal system. Menzamine A and other menzamines have shown a variety of biological activities, including antitumor, antimalarial, antimicrobial, insecticidal, anti-inflammatory, and immunosuppressive [373]. Manzamine-related alkaloids lissodendoric acids A and B isolated from the marine sponge *Lissodendoryx florida* from the Sea of Okhotsk have revealed unique structural features, including a conjugated carboxydiene moiety, a longer saturated intramolecular hydrocarbon chain, and an unprecedented (CH_2_)_10_NH_2_ substituent instead of the conventional manzamine-bridged system. In an in vitro model of PD based on Neuro2a cells treated with 6-hydroxydopamine, lysodendoric acids A and B have shown a potent ability to reduce ROS and promote cell survival [374].

The β-carboline alkaloid fascaplysin, originally isolated from the Fijian sponge *Fascaplysinopsis* sp., inhibited AChE and induced the expression and activity of P-glycoprotein [375]. Subsequent studies demonstrated fascaplysin could inhibit the formation of Aβ42 fibrils in vitro. At the same time, the synthetic analog 9-methylfascaplysin reduced the formation of Aβ oligomers by interacting with negatively charged Aβ42 residues, attenuating the toxic effect of Aβ42 on SH-SY5Y cells [376]. At the PIBOC center (Vladivostok), routes for chemical synthesis of fascaplysin and its derivatives 6-oxofascaplysin, homofascaplysins B, 3-bromohomofascaplysin B, 3-bromohomofascaplysin B-1, and 3,10-dibromofascaplysin have been developed for subsequent assessment of their biological activities including those targeting AD [377,378,379].

The main representatives of bioactive compounds from marine organisms from the Russian Pacific discussed above are summarized in Table 1:

## 4. Discussion

The data and findings we have presented provide examples of promising anti-neurodegenerative marine compounds originating from animals, algae, microorganisms, and fungi of the Russian Pacific, acting at different targets relevant to the anti-NDD pharmacology. Despite the impressive research achievements behind them, it is clear that these examples are just ‘scrapes of the surface’ of the pharmacological potential of the region. Indeed, the Russian Pacific is estimated to represent a treasury of up to 1 million unique marine compounds, with only a minuscule share of >500 compounds being characterized so far [21,380]. Of >30,000 marine compounds discovered globally [381], >130 have revealed biomedical properties with neurology indications in preclinical settings [382,383,384,385,386,387,388,389,390,391]. Of those, three marine compounds are currently in clinical trials for neurology/NDD applications, and one is currently FDA-approved [392]. Projected on the estimates of the marine chemodiversity of the Russian Pacific, we may expect some 5000 unique compounds to emerge from the intense elaboration of the marine pharmacology potential of the Russian Pacific, of which >100 to enter clinical trials as first-in-class treatments against NDD [21]. The global NDD treatment market is currently estimated at ca. 40 billion USD. It is estimated to grow by 3.1% annually [393], and marine-based drug discovery can take a significant share in this value. With the number of individuals above 60 affected by NDD expected to amount to 2.1 billion by 2050 [393], NDD has become the curse of aging humanity. The fight against them becomes of paramount importance, and all the arsenals should be considered in this ‘war on NDD’. Large-scale exploration of the marine pharmacology potential of the Russian Pacific could and should play an important part in this challenge.

## Figures and Tables

**Table 1:** Taxonomy of marine organisms from the Russian Pacific and their bioactive compounds with experimentally confirmed anti-neurodegenerative activity.

Species ^1^	Bioactive Compounds	Pharmacological Effect/Mechanism	Ref.
*Laminaria digitata* and other Phaeophyceae, phylum Ochrophyta	Laminarans	Reduction of expression on proinflammatory cytokines; increase of expression of SOD and anti-inflammatory cytokines; neuroprotective effects	[325,327,328]
*Saccharina cichorioides*, *Fucus evanescens*, and other Phaeophyceae, phylum Ochrophyta	Fucoidans	Inhibition of BACE1; suppression of Aβ production and aggregation; decrease in Aβ42 toxicity; decrease in ROS production; decrease in tau hyperphosphorylation; inhibition of caspase-9 and caspase-3 activation; neuroprotective effects	[329,330,331,332,335,336,337,338,339,340,341]
*Pelvetia siliquosa*, *Eisenia bicyclis*, and other brown algae	Fucosterol, other sterols	Increase of expression of SOD, GPX1, and CAT; inhibition of ROS production; increase in glutathione levels; inhibition of AChE and BChE; suppression of Aβ aggregation and neuroinflammation	[284,285,286,289,294,298]
*Saccharina gurjanovae, Alaria marginata*, *Cystoseira crassipes*, *Saccharina bongardiana*, *Arthrothamnus bifidus*, *Eualaria fistulosa*, *Fucus evanescens*, and other Phaeophyceae, phylum Ochrophyta	Phlorotannins	Antioxidant and anticholinesterase activities; potential inhibition of the Wnt signaling pathway	[315,316,318,320,321,322,324]
*Sargassum horneri* and other Phaeophyceae, phylum Ochrophyta	Fucoxanthin	Decrease in Aβ aggregation; decrease in the formation of Aβ oligomers and fibrils; increase in glutathione and SOD activity; increase in ChAT activity; suppression of microglia activation; increase in expression of BDNG; decrease in levels of proinflammatory mediators; decrease in expression of iNOS and COX-2; decrease in MAPK signaling; activation of PI3K/Akt signaling	[344,345,346,347]
Starfish *Luidia maculate*, *Patiria pectinifera*, *Linckia laevigata*	Sphingolipids	Neuritogenic activity	[274]
Holoturians *Stichopus japonicus*, *Stichopus chloronotus*, *Cucumaria echinate*	Sphingolipids	Neuritogenic activity	[274]
Sponge *Inflatella* sp.	Oxysterols	Inhibition of BACE1; reduction in ROS production; increase in neuronal survival; neuroprotective effects	[312,314]
Sea urchin *Scaphechinus mirabilis*	Echinochrome	Antioxidant and neuroprotective effects	[353,355,356,357]
Starfish *Hippasteria kurilensis*, *Linckia laevigata*, *Aphelasterias japonica*, *Leptasterias ochotensis*, *Linckia hylodes reticulata*, *Lethasterias fusca*, *Lethasterias nanimensis chelifera*, *Aphelasterias japonica*	Asterosaponines	Anti-inflammatory, neuritogenic, and neuroprotective effects	[359,360,361,362,363,364,365,366,367,368,369,370]
Sponge *Lissodendoryx florida*	Lissodendoric acids A and B	Decrease in ROS	[374]
Sponge *Fascaplysinopsis* sp.	Fascaplysin and its synthetic analogs	Inhibition of AChE; inhibition of Aβ42 fibrils	[377,378,379]
Crabs *Paralithodes camtschaticus, Paralithodes platypus, Chionoecetes opilio, Chionoecetes angulatus, Chionoecetes japonicus*	Docosahexaenoic acid, eicosapentaenoic acid	Reduction in tau phosphorylation; reduction in the levels of proinflammatory cytokines; prevention of neuroinflammation; antioxidant effects; activation of remyelination; improvement of motor and cognitive functions; neuroprotective effects	[220,239,244]
Hepatopancreas of crab *Paralithodes camtschaticus*, liver of squid *Berryteuthis magister*, liver of stingray *Bathyraja parmifera*	1-O-alkyl-glycerols	Reduction in expression of proinflammatory cytokines; prevention of activation of M1 microglia	[230,233,235]
*Oncorhynchus gorbuscha* (and other pacific salmon)	N-docosahexanoylethanolamine	Suppression of TNF-α expression, NO production, and neuroinflammation; activation of synaptogenesis; neuroprotective effects	[252,253,257]
Pacific saury *Cololabis saira*	Phosphatidylcholine, phosphatidylinositol, phosphatidylethanolamine	Suppression of Aβ42 release	[259]

^1^ Marine organisms’ classification is presented in accordance with the World Register of Marine Species.

## Data Availability

Not applicable.
